# The ALDH Family Contributes to Immunocyte Infiltration, Proliferation and Epithelial-Mesenchymal Transformation in Glioma

**DOI:** 10.3389/fimmu.2021.756606

**Published:** 2022-01-07

**Authors:** Zeyu Wang, Yuyao Mo, Ying Tan, Zhihui Wen, Ziyu Dai, Hao Zhang, Xun Zhang, Songshan Feng, Xisong Liang, Tao Song, Quan Cheng

**Affiliations:** ^1^ Department of Neurosurgery, Xiangya Hospital, Central South University, Changsha, China; ^2^ National Clinical Research Center for Geriatric Disorders, Xiangya Hospital, Central South University, Changsha, China; ^3^ Clinic Medicine of 5-Year Program, Xiangya School of Medicine, Central South University, Changsha, China; ^4^ Department of Blood Transfusion, Xiangya Hospital, Central South University, Changsha, China; ^5^ Clinical Diagnosis and Therapy Center for Glioma of Xiangya Hospital, Central South University, Changsha, China; ^6^ Department of Clinical Pharmacology, Xiangya Hospital, Central South University, Changsha, China

**Keywords:** ALDH, gliomas, proliferation, migration, immunocytes

## Abstract

Gliomas are malignant tumors that originate from the central nervous system. The aldehyde dehydrogenase family has been documented to affect cancer progression; however, its role in gliomas remains largely unexplored. Bulk RNA-seq analysis and single-cell RNA-Seq analysis were performed to explore the role of the aldehyde dehydrogenases family in gliomas. Training cohort contained The Cancer Genome Atlas data, while data from Chinese Glioma Genome Atlas and Gene Expression Omnibus were set as validation cohorts. Our scoring system based on the aldehyde dehydrogenases family suggested that high-scoring samples were associated with worse survival outcomes. The enrichment score of pathways were calculated by AUCell to substantiate the biofunction prediction results that the aldehyde dehydrogenases family affected glioma progression by modulating tumor cell proliferation, migration, and immune landscape. Tumor immune landscape was mapped from high-scoring samples. Moreover, ALDH3B1 and ALDH16A1, two main contributors of the scoring system, could affect glioblastoma cell proliferation and migration by inducing cell-cycle arrest and the epithelial-mesenchymal transition. Taken together, the aldehyde dehydrogenases family could play a significant role in the tumor immune landscape and could be used to predict patient prognosis. ALDH3B1 and ALDH16A1 could influence tumor cell proliferation and migration.

## Introduction

Gliomas are the most common cancer type of the central nervous system, representing approximately 80% of malignant brain tumors. Glioblastoma (GBM) is the most aggressive type of gliomas (1, 2), with a reported overall survival time of only 15-18 months (3, 4). However, the current classification based on histology has major limitations and cannot precisely predict patient outcomes (5). Over the past decades, the identification of molecular signatures has provided new insights into glioma prognosis prediction (6). For instance, isocitrate dehydrogenase (IDH) 1/2 mutations and 1p19q co-deletion have been considered glioma prognosis-related biomarkers (7). Interestingly, classification based on these signatures can be used to subdivide GBM into three subtypes (classical, proneural and mesenchymal) and assist during clinical practice for treatment selection and prognosis prediction (8). Since gliomas are highly heterogeneous tumors, it is vital to identify novel biomarkers to help clinicians during the differential diagnosis and make treatment decisions.

Aldehyde dehydrogenases (ALDHs) are a group of enzymes that catalyze the oxidation of aldehydes to less toxic carboxylic acids (9). The human ALDH superfamily consists of 19 putative members distributed in different organs. Multiple studies reported that ALDHs were associated with tumorigenesis and tumor resistance to therapy. In this regard, some ALDHs have been discovered to be biomarkers of cancer stem cells (10, 11). For instance, ALDH1A3 has been reported to participate in extracellular matrix reorganization and cell adhesion in gliomas by inducing mesenchymal transformation (12–14) and is responsible for the aggressiveness of mesenchymal-like glioma stem cells (15, 16). Furthermore, ALDH1A1 has been associated with glioblastoma proliferation and migration (17, 18), while decreased ALDH1A1 expression could restore GBM cell sensitivity to temozolomide, a common and efficient chemotherapeutic drug for GBM (19). Accordingly, the association between ALDHs and cancer is complicated, warranting further studies.

In the present study, the expression profile of ALDHs in gliomas and their different clinical features were explored. A scoring system based on the least absolute shrinkage and selection operator (LASSO) regression analysis calculated the risk score for each sample. The high-risk group showed worse overall survival outcomes relative to the low-risk group. Besides, GO/KEGG enrichment analysis and AUCell analysis from single-cell RNA-Seq analysis corroborated that ALDHs promoted glioma progression by affecting cell proliferation and migration. Notably, we found that two contributors of that system, ALDH3B1 and ALDH16A1, could induce cell cycle arrest at the G2/M phase and epithelial-mesenchymal transition by performing a CCK8 assay, colony-forming assay, transwell assay and flow cytometry analysis. Finally, a prognostic risk score model was established to assist in clinical decision-making.

## Materials And Methods

### mRNA Data Acquisition and Processing

mRNA data were downloaded from public databases, including The Cancer Genome Atlas (TCGA), Chinese Glioma Genome Atlas (CGGA) and Gene Expression Omnibus (GEO). Samples were separated as training (TCGA dataset) and validation cohorts (CGGA dataset and GSE108474 dataset); CGGA sequencing data and microarray data were termed CGGA1 and CGGA2 datasets, respectively. Detailed information is listed in **Supplementary Table 1**.

mRNA data for Single-cell RNA-Seq analysis was downloaded from the GSE139448 dataset (20). GSE139448 dataset consisted of three GBM samples, GBM27, GBM28 and GBM29. R packages “Seurat”, “NormalizeData” and “FindVariableGenes” were applied to normalize the expression matrix and identify markers for each cell type. Cluster analysis was performed using PCA and visualized with the UMAP algorithm. Six cell types were clustered, including neoplastic (tumor cells), oligodendrocyte progenitor cells (OPC), macrophage, vascular, oligodendrocyte and NK cells. Among these cell types, OPC, vascular and oligodendrocyte were defined as tumor stromal cells.

### Single Nucleotide Polymorphisms and Copy Number Variations

The single nucleotide polymorphisms (SNPs) and copy number variations (CNVs) were generated by performing GISTIC 2.0 (21). The nomogram and corresponding ROC curve were constructed.

### The Risk System and Biofunction Prediction

A risk score of each sample was calculated by LASSO regression analysis as described in our previous work (22). High and low-risk groups were separated based on the median value of the risk score. Genes enriched in three GO pathways: GO_REGULATION_OF_EXTRACELLULAR_MATRIX_DISASSEMBLY, GO_REGULATION_OF_CELL_CYCLE_CHECKPOINT and GO_T_CELL_MEDIATED_IMMUNITY were downloaded from http://www.gsea-msigdb.org/gsea/msigdb. The correlation between contributors of the scoring system and genes involved in those three pathways was calculated using the Pearson correlation coefficient.

Then, we examined the activation of those pathways in GBM samples with single-cell RNA-seq analysis. GO enrichment analysis and the AUC score of three GO pathways, including T cell-mediated immunity, regulation of cell cycle arrest and extracellular matrix disassembly, were performed by using the “clusterProfiler” and “AUCell” package, respectively (23, 24). Only tumor cells were analyzed for this part.

### Tumor Immune Landscape

The immune landscape of glioma between two groups was evaluated by the ESTIMATE algorithm (25), the CIBERSORT algorithm (26) and 28 subpopulations of immunocytes (27) with R packages “ESTIMATE”, “CIBERSORT” and “ssGSEA”. The expression profile of tumor immune escape-related genes was also illustrated (28, 29).

### Cell Culture and Transfection

Human glioma cells, U251 and U87MG, were purchased from the Chinese Academy of Sciences. Cells were cultured in a DMEM medium with 10% fetal bovine serum and 1% penicillin-streptomycin. Cells were subdivided into the control group, the siRNA negative control group (the si-NC group), the transfection group (ALDH3B1: siRNA-462, siRNA-1155; ALDH16A1: siRNA-1192, siRNA-536). The siRNA was diluted to 100nM for transfection, using Lipofectamine^®^ 2000 Reagent according to the manufacturer’s protocol.

### Real-Time Quantitative PCR

RNA was extracted using Trizol reagent. Agarose gel electrophoresis was used to demonstrate the high integrity of extracted RNA. The cDNA was then synthesized and added to a PCR mix along with forward and reverse primers of each gene. The PCR mix consisted of 2 ul cDNA, 10 ul SYBR Green, 0.8 ul of the former primer, 0.8 ul latter primer and 6.4 ul ddH2O, as the description of TSINGKE Master qPCR Mix (Tsingke Biotechnology Co., Ltd., Beijing). The primers were designed by primer5 and synthesized by Sangon Biotech (Shanghai, China). The cycling conditions were set as follows: 95°C for the first 10 minutes, followed by 40 cycles at 95°C for 15 seconds and, finally, 60°C for 30 seconds. The primer sequences used are listed below:

β-actin: Forward: ACCCTGAAGTACCCCATCGAG; Reverse: AGCACAGCCTGGATAGCAAC

ALDH3B1: Forward: GTTTCCTGCCTGACAACCCTT; Reverse: AGCAGCCTCTCATTGTCCA

ALDH16A1: Forward: CCTGAGCGCCACCTACTGCAT; Reverse: CTGCCAGAAGTTGCTACACCCTT

### Western-Blot Assay

DTT and PMSF were added to RIPA before lysing cells and the supernatant was collected after cell lysis with RIPA (Shanghai, China). The protein concentration was determined by the bicinchoninic acid method. Samples were run on 10% SDS-PAGE gel at 75mV. The bolts were transformed to membrane at 300mA. The membrane was incubated with 5% non-fat milk for 2 hours, and then primary antibodies were added. Primary antibodies including ALDH3B1 (1:1000, Proteintech, Cat# 19446-1-AP, RRID: AB_2861362), ALDH16A1 (1:2000, Abcam, Cat# ab137082, RRID: AB_2861363), E-cadherin (1:5000, Proteintech Cat# 20874-1-AP, RRID : AB_10697811), N-cadherin (1:1000, Abcam Cat# ab76011, RRID : AB_1310479), snai1 (1:1000, Proteintech Cat# 13099-1-AP, RRID : AB_2191756), β-catenin (1:5000, Proteintech Cat# 51067-2-AP, RRID : AB_2086128), cyclin A (1:2000, Proteintech Cat# 18202-1-AP, RRID : AB_10597084), cyclin B1 (1:2000, Proteintech Cat# 55004-1-AP, RRID : AB_10859790), aurora A (1:500, Proteintech Cat# 10297-1-AP, RRID : AB_2061337) and β-actin (1:5000, Proteintech Cat# 66009-1-Ig, RRID: AB_2687938) were diluted by antibody dilution buffer.

On the second day, the membrane was incubated with horseradish peroxidase-conjugated secondary antibodies (1:2000, Proteintech Cat# SA00001-1, RRID: AB_2722565; 1:2000, Proteintech Cat# SA00001-2, RRID: AB_2722564) for 1 hour at room temperature. Bands were visualized using enhanced chemiluminescence (ECL) solution, and pictures were captured by AI600 Chemiluminescent Imager (Cytiva, USA).

### CCK8 Assay

Cells were plated into 96-well plates at a concentration of 1×10^3^ cells per well. Then, cells were cultured at 37°C with 5% CO2. The OD value of 450nm was measured at 0h, 12h, 24h, 48h and 72h after adding culture medium containing 10% cck8 (Dojindo, Japan).

### Transwell Assay

100 μl single-cell suspensions (1x106 cells per milliliter) with empty DMEM were added to the upper chamber (Corning), while DMEM with 20% bovine serum was added to the lower chamber. After incubating cells at 37°C with 5% CO2 for 48 hours, the chamber was washed with PBS and cells on the upper chamber side surface of the membrane were removed with a cotton swab. Cells attaching to the lower surface were fixed and stained. The OD value at 550nm was measured after cell discoloration with acetic acid.

### Flow Cytometry Assay

1×10^6^ cells per milliliter single-cell suspensions were collected and fixed with pre-cooled 75% ethanol. Each sample was stained with Propidium Iodide Solution consisting of 500ul PI stain buffer, 25ul PI stain and 10ul RNase A Solution. After incubating cells at 37°C in the dark for 30 minutes, samples were uploaded on a flow cytometer. The percentage of cells in the G0, G1 and S phases were analyzed using ModFit LT (version 5.0).

### Colony-Forming Assay

Cells were digested with 0.25% trypsin, then were plated into a 6-well plate at a concentration of 200 cells per well. Afterward, cells were incubated in cell incubators for 14 days. Then, 4% methanol was applied to fix the cells colony, and crystal violet was used to stain those colonies. The OD value at 550nm was measured after cell discoloration.

### Statistical Analysis

Differences between the two groups were analyzed by the Mann-Whitney U test, while one-way ANOVA analysis was conducted for multiple groups. A two-way ANOVA analysis was conducted to ananlyze the CCK8 assay. Kaplan-Meier survival curves were generated and compared by using the log-rank test. Statistical analysis for the colony-forming assay, the CCK8 assay and the transwell assay were carried out by GraphPad Prism (version 8.0). A P-value < 0.05 was considered to be statistically significant. All bioinformatics analyses were conducted by R (version 3.6.2).

## Results

### Abnormal ALDH Expression Was Associated With Gliomas Pathological Grades

TCGA data (522 lower-grade glioma (LGG) samples and 150 GBM samples) was used as the training cohort, while the CGGA1 (180 LGG samples and 136 GBM samples), CGGA2 (173 LGG samples and 124 GBM samples) and GSE108474 (170 LGG samples and 124 GBM samples) datasets were set as validation cohorts.

**Figure 1:**
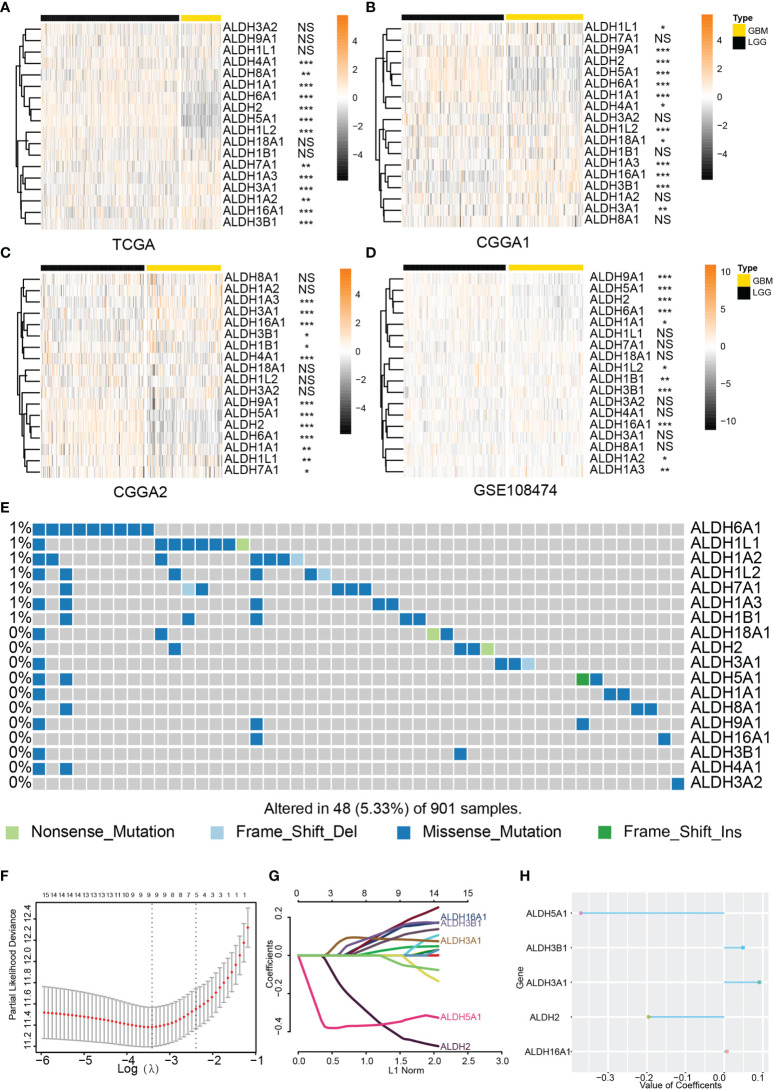
Expression profile of the ALDH family. The ALDH family expression of normal brain tissue and glioma. The expression profile of ALDHs between WHO grades of glioma from the TCGA **(A)**, CCGA1 **(B)**, CGGA2 **(C)** and GEO **(D)** database. **(E)** Genomic alteration of ALDHs in glioma. OPC: oligodendrocyte precursor cell. **(F–H)** The construction of the scoring model based on the LASSO algorithm. NS, no significantly statistical; *P < 0.05; **P < 0.01; ***P < 0.001.

The correlation between ALDH expression and clinicopathological parameters of gliomas, including pathological glioma grade and IDH status, was first investigated. Analysis of the TCGA dataset showed higher expression levels of ALDH16A1, ALDH3B1, ALDH7A1, ALDH1A2, ALDH3A1 and ALDH1A3 in GBM compared to LGG, while ALDH4A1, ALDH8A1, ALDH1A1, ALDH6A1, ALDH2, ALDH5A1, ALDH1L2 expressions were lower in GBM (**Figure 1A**). In the validation cohorts, lower expressions of ALDH1A1, ALDH6A1, ALDH2, ALDH5A1 were observed in GBM compared to LGG, while ALDH16A1, ALDH3B1 and ALDH3A1 were preferentially enriched in GBM (**Figures 1B–D**).

In the LGG cohort from the TCGA dataset, ALDHs including ALDH2, ALDH6A1, ALDH5A1, ALDH9A1, ALDH1A1 were upregulated in WHO grade II gliomas while the increased expression of ALDH16A1, ALDH3B1, ALDH3A2, ALDH1A2, ALDH3A1 was found in WHO III grade gliomas (**Supplementary Figure 1A**). Similar alterations of ALDH2, ALDH6A1, ALDH5A1, ALDH1A1, ALDH16A1, ALDH3B1, ALDH3A2, ALDH3A1 expression were observed in CGGA and GEO108474 validation datasets (**Supplementary Figures 1B–D**).

The IDH status was closely associated with glioma progression, and IDH mutant gliomas had better clinical outcomes. Analysis of the TCGA dataset showed that ALDH16A1, ALDH3B1, ALDH1A3, ALDH3A1, ALDH7A1 were significantly increased in IDH wildtype gliomas, while ALDH2, ALDH6A1, ALDH5A1, ALDH1A1, ALDH1L2, ALDH18A1, ALDH1B1 expression were higher in IDH mutant gliomas than IDH wildtype gliomas (**Figure S1E**). Similar gene expression dysregulation between IDH wildtype and IDH mutant gliomas were verified in validation datasets (**Supplementary Figures 1F, G**).

Moreover, the incidence of somatic ALDH mutations in gliomas was 5.33% (48/901), and most of them were missense mutations. This result further substantiated the presence of ALDH dysregulations in gliomas (**Figure 1E**). Taken together, the expression of most ALDHs was altered, and this dysregulation was associated with glioma progression.

### The Risk Scoring System Can Predict Glioma Patient Prognosis

To explore the prognostic value of different ALDHs in glioma patients, univariate Cox regression was applied to assess the relationship between individual ALDHs and the overall survival outcome of glioma patients from the TCGA dataset. A total of 16 ALDHs was significantly associated with overall survival (OS) with p-values less than 0.05 (**Supplementary Table 2**). ALDH2, ALDH5A1, ALDH6A1, ALDH1L2, ALDH1B1, ALDH18A1, ALDH1A1, ALDH8A1, ALDH1B1 were found to be favorable factor for glioma patients with a hazard ratio (HR) <1. ALDH16A1, ALDH3B1, ALDH3A1, ALDH7A1, ALDH1A3, ALDH1L1, ALDH1A2 were risk genes with HR > 1. Moreover,overall survival analysis based on ALDHs further confirmed their prognostic roles in gliomas (**Supplementary Figure 2**).

**Figure 2:**
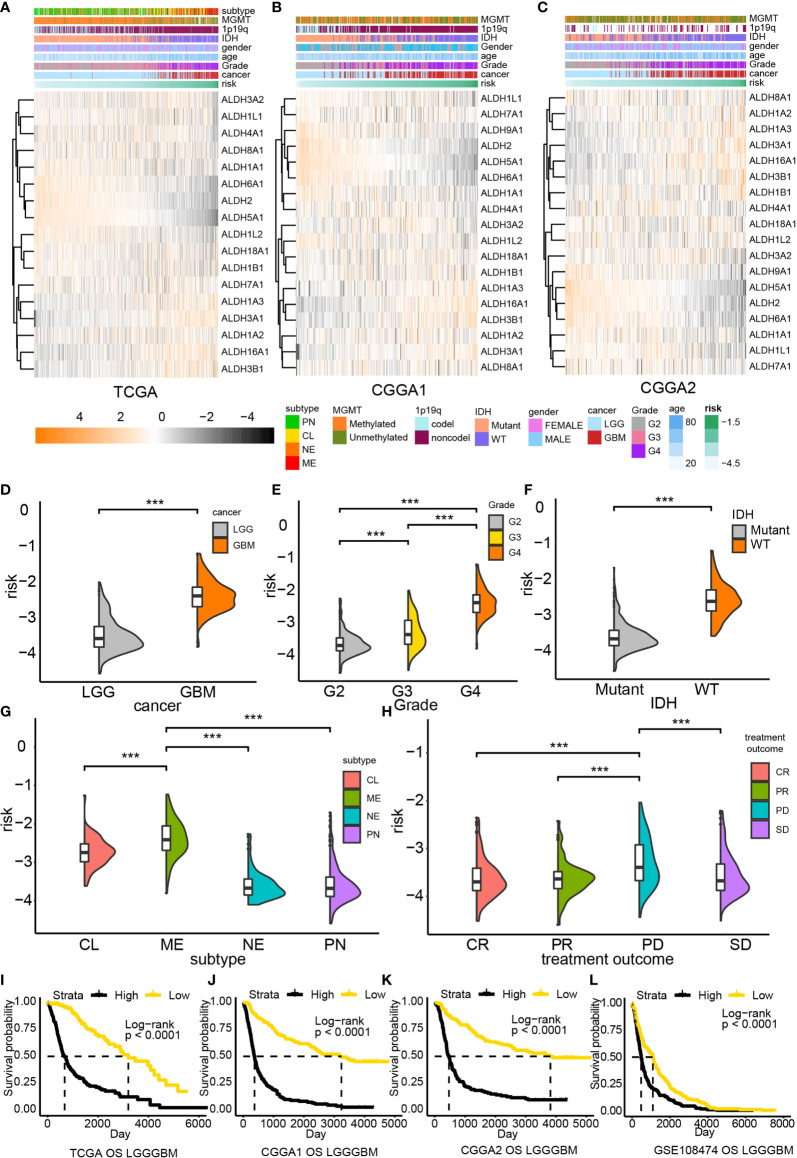
The risk model based on the ALDH family. Association between ALDHs expression and risk in TCGA **(A)**, CCGA1 **(B)** and CGGA2 **(C)** database. The distribution of risk in gliomas grades **(D, E)**, the IDH status gliomas **(F)**, tumor subtype **(G)** and tumor response to treatment **(H)**. Overall survival analysis based on risk in TCGA **(I)**, CCGA1 **(J)** CGGA2 **(K)** and GEO **(L)** database. G2, grade II; G3, grade III; CL, classical; ME, mesenchymal; NE, neural; PN, proneural; CR, complete remission/response; PR, partial remission/response; PD, progression diseases; SD, stable diseases. ***P < 0.001.

Subsequently, a risk model based on the LASSO regression analysis was constructed, which consisted of five genes (**Figures 1F–H**). The risk score for each sample was calculated based on these genes. The performance of our risk model was also assessed using data from the validation cohort. Heatmaps were built to illustrate the correlation between the risk score and ALDHs expression (**Figures 2A–C**). We found that the expression of ALDH2 and ALDH5A1 were inversely correlated with the risk score, while ALDH3A1, ALDH 3B1 and ALDH16A1 expression positively correlated with the risk score.

The risk score distribution for different clinical features was also analyzed. High-risk samples were associated with malignant glioma features, including high-grade (**Figures 2D, E**), IDH wildtype (**Figure 2F**), and classical or mesenchymal (**Figure 2G**). Besides, high-risk samples also exhibited worse treatment responses than low-risk samples (**Figure 2H**). A similar tendency could also be noticed in validation datasets (**Supplementary Figure 3A**). Taken together, high-risk samples were associated with worse overall survival outcomes than low-risk samples.

Subsequently, the prognostic value of different risk scores obtained from glioma patients was evaluated by performing survival analysis. High risk patients were strongly associated with poor clinical outcome in TCGA (P < 0.0001, **Figure 2I**), CGGA1 (P < 0.0001, **Figure 2J**), CGGA2 (P < 0.0001, **Figure 2K**) and GSE108474 datasets (P < 0.0001, **Figure 2L**). Moreover, our risk model exhibited a good performance in predicting survival outcomes of LCG patients but performed poorly for GBM patients (**Supplementary Figure 3B**). Nevertheless, a significant difference in the survival outcome of GBM patients in the CGGA1 (P = 0.025) and CGGA2 (P = 0.027) cohorts was found; a similar tendency was noticed in the GSE108474 dataset; however, the difference was not statistically significant. Therefore, the poor predictive performance of our risk model could result from the limited number of GBM samples.

### Somatic Mutations and Copy Number Variations Between High and Low-Risk Groups

**Figure 3:**
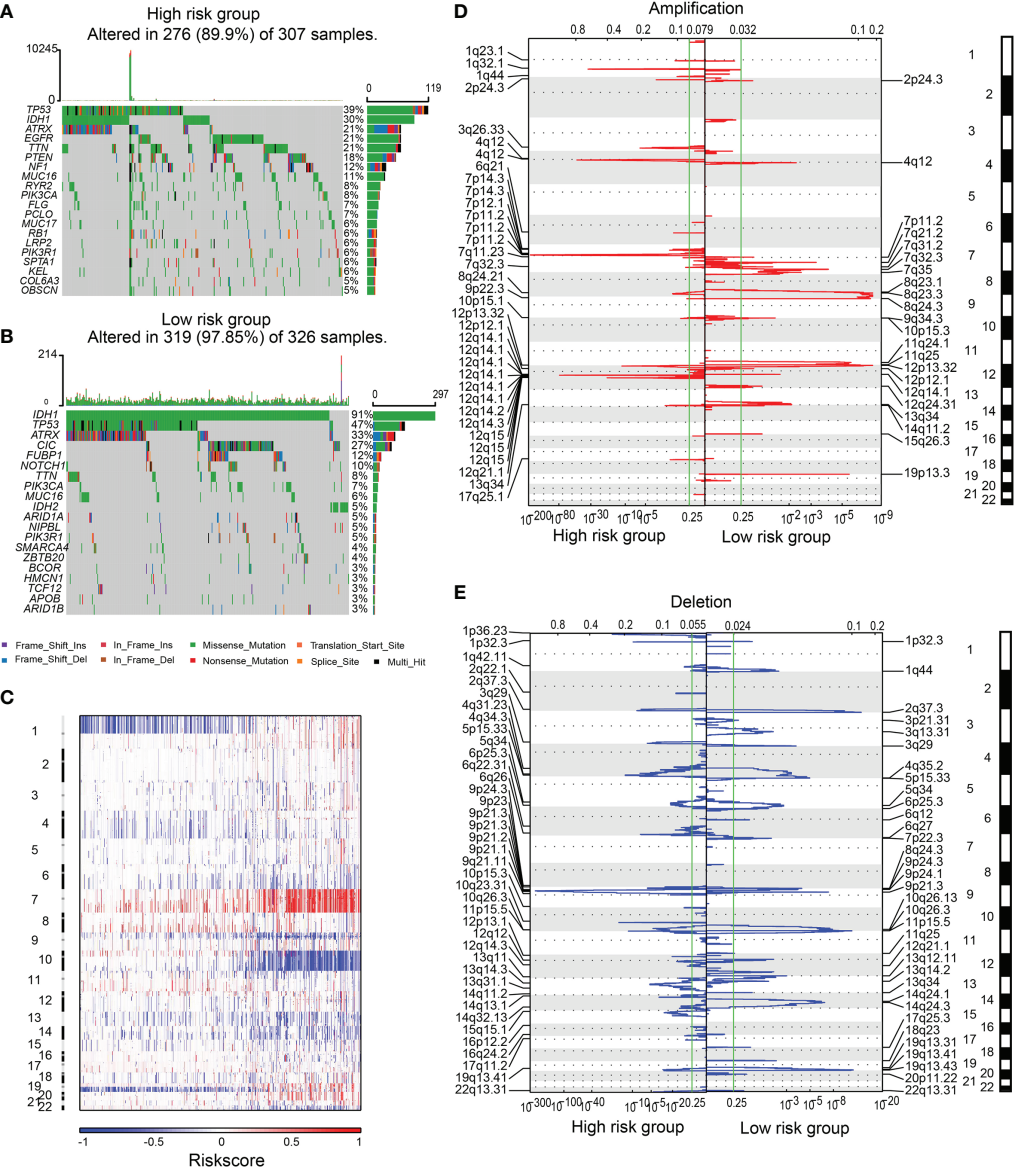
The SNPs and CNVs between high and low-risk groups. SNPs in high **(A)** and low-risk **(B)** groups. **(C)** The overview of the incidence of CNVs ranked by risk. Amplification **(D)** or deletion **(E)** regions in samples from high or low-risk group.

Next, we explored the difference in somatic mutations and copy number variations between high and low-risk groups. The overall mutation ratio was 89.9% and 97.85% in groups with high and low-risk scores. A higher frequency of IDH1, TP53, ATRX mutation was found in the low-risk group than in the high-risk one (IDH1 91% *vs*. 30%, TP53 47% *vs*. 39%, ATRX 33% *vs*. 21%). In contrast, a lower mutation frequency of TTN, MUC16, and PIK3CA was found in the high-risk group than in the low-risk group (TTN 8% *vs*. 21%, MUC16 6% *vs*. 11%, PIK3CA 7% *vs*. 8%). In addition, mutations of CIC (27%),FUBP1 (12%), and NOTCH1 (10%) were observed in the lowriskgroup, while EGFR (21%), PTEN (18%), and NF1 (12%) mutations were identified in the high-risk score group (**Figures 3A, B**).

CNVs analysis revealed that glioma patients with high-risk scores featured frequent amplification of Chr 7 and deletion of Chr 9 and Chr 10 (**Figure 3C**). GISTIC 2.0 analysis revealed that in gliomas with high-risk scores, the amplification mainly occurred in chromosomal regions 1q32.1, 3q26.33, 4q12, 7p11.2, and 12q14.1, where several key oncogenes (PDGFRA, EGFR, MDM2, SOX2, and CDK4) were located. Focal deletions in chromosomal regions 9p21.3 and 10q23.31, which encode tumor suppressors including PTEN, FAS, and CDKN2A, were observed (**Figures 3D, E**).

### The Clinical Application of the Risk Scoring System

Independent prognostic indicators were identified by employing univariate and multivariate Cox analysis (**Supplementary Figure 4**). Schoenfeld individual tests were performed to identify biomarkers for the construction of nomogram (**Supplementary Figure 5A**). A nomogram was built to predict the survival of patients using those variables, and each variable was assigned a score (**Supplementary Figure 5B**). Calibration curves proved that this model could precisely predict a patient’s survival outcome (**Supplementary Figure 5C**). The overall survival analysis suggested shorter survival of patients with high nomogram scores than patients with low nomogram scores. Similar results were obtained in the CGGA datasets (**Supplementary Figures 5D–F**). The area under the curve (AUC) of receiver operating characteristic (ROC) curves predicting the overall survival probability at 3 and 5 years was 0.882 and 0.816, respectively, in the TCGA dataset. Validation results from the CGGA1 (AUC at 3 years OS: 0.876; AUC at 5 years OS: 0.890) and CGGA2 (AUC at 3 years OS: 0.881; AUC at 5 years OS: 0.812) also substantiated this conclusion (**Supplementary Figures 5G–I**).

### ALDHs May Affect Tumor Immune Landscape, Tumor Proliferation and Migration

**Figure 4:**
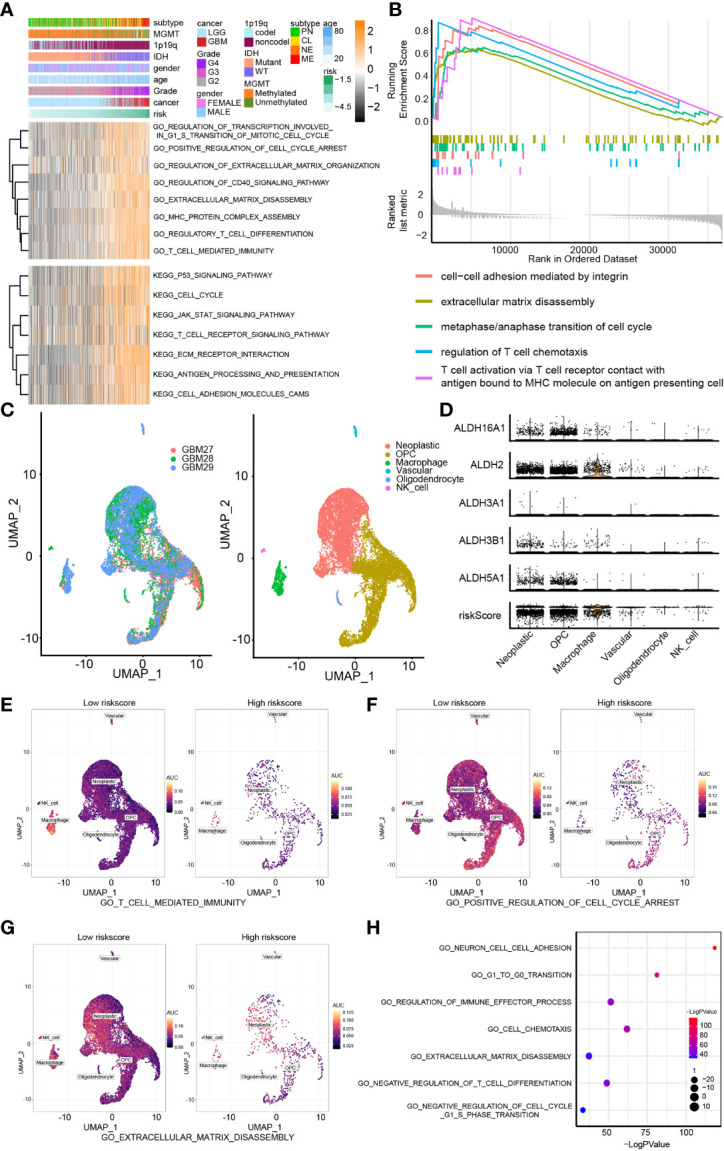
Biofunction prediction based on bulk RNA-Seq analysis and single-cell analysis. **(A)** The GO and KEGG enrichment analysis based on the GSVA algorithm. **(B)** The GO enrichment analysis based on the GSEA algorithm. **(C)** The cell components of single cell RNA-seq analysis. **(D)** The distribution of ALDH2, ALDH16A1, ALDH3B1, ALDH5A1, ALDH3A1 and risk in different cell components. The AUC score of three pathways, including T cell mediated immunity **(E)**, positive regulation of cell cycle arrest **(F)** and extracellular matrix disassembly **(G)**, from the GO enrichment analysis were calculated. **(H)** The GO enrichment analysis based on single-cell analysis.

GSVA analysis was applied to perform GO and KEGG enrichment analysis (**Figure 4A**). Pathways related to cell cycle, cell adhesion and tumor immune landscape were enriched in the high-risk group, including regulation of transcription involved in G1/S transition of the mitotic cell cycle, cell cycle, extracellular matrix disassembly, regulation of extracellular matrix organization, T-cell mediated immunity, regulation of T cell chemotaxis, regulatory T cell differentiation. The GSEA findings were consistent with the GO/KEGG enrichment analysis results (**Figure 4B**).

**Figure 5:**
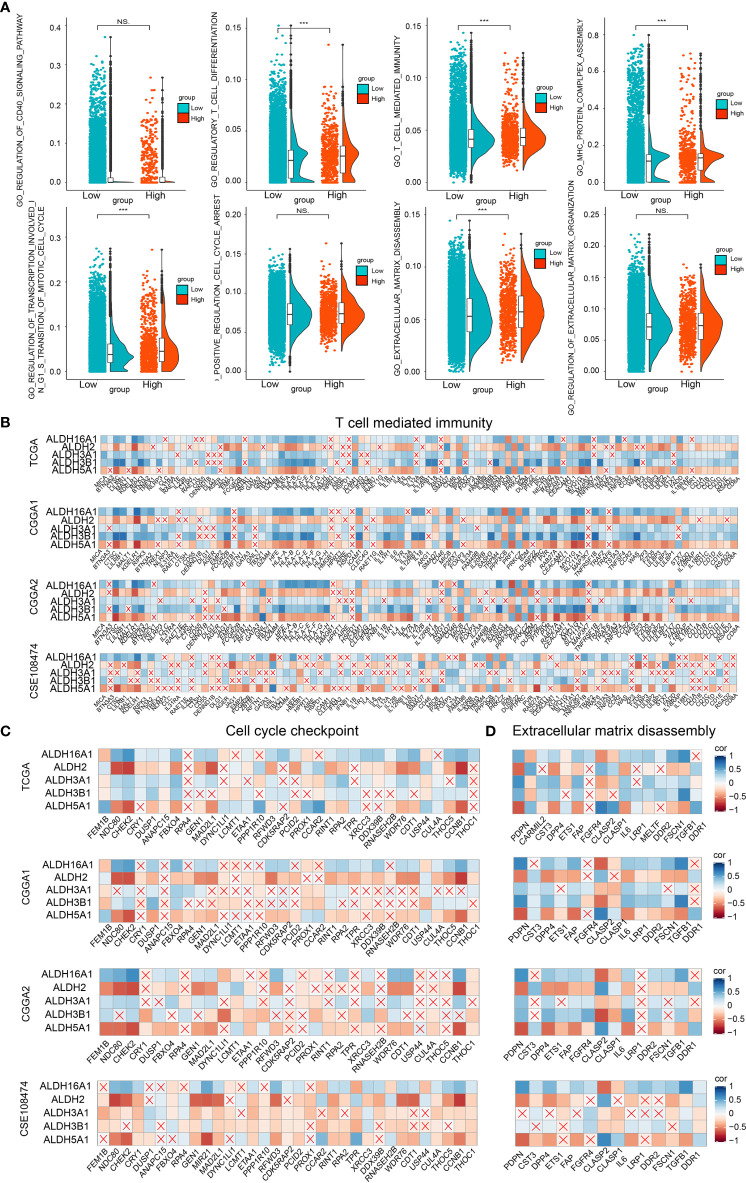
Biofunction prediction based on bulk RNA-Seq and single-cell RNA-Seq analysis. **(A)** The enrichment score distribution of GO pathways based on single-cell analysis. Association between contributors of the scoring system and genes enriched in T cell mediated immunity **(B)**, cell cycle checkpoint **(C)** and extracellular matrix disassembly **(D)** in TCGA, CGGA1, CGGA2 and GSE108474 datasets. NS, no significantly statistical; ***P < 0.001.

Moreover, we examined the relevance of these pathways with pure tumor cells using single-cell RNA-Seq analysis. Six cell components were identified, including tumor cells, OPC, macrophage, vascular, oligodendrocyte and NK cells (**Figure 4C**). The distribution of the components of our risk score model and risk score associated with these cell components was mapped; and ALDHs were mostly enriched in neoplastic cells and macrophage (**Figure 4D**). The activation of three GO pathways, T cell-mediated immunity (**Figure 4E**), positive regulation of cell cycle arrest (**Figure 4F**) and extracellular matrix disassembly (**Figure 4G**), were evaluated by calculating the AUC score. Importantly, results from the GO enrichment analysis of single-cell RNA-seq based on R package “clusterProfiler” (**Figure 4H**) and “GSVA” both proved those results (**Figure 5A**).

To improve our understanding of the relationship between components of our risk model and the tumor immune landscape, cell cycle and migration, the correlation between different ALDHs and genes from GO pathways, including T cell-mediated immunity (**Figure 5B**), cell cycle checkpoint (**Figure 5C**) and extracellular matrix disassembly pathways (**Figure 5D**) was analyzed. ALDH16A1, ALDH3A1, ALDH3B1 positively correlated with genes in these pathways, while the opposite finding was observed for ALDH2 and ALDH5A1. Thus, these results suggested that ALDHs could affect the tumor immune landscape, tumor cell cycle and migration.

### ALDHs Can Affect Immunocyte Infiltration and the Expression of Immune Checkpoints

**Figure 6:**
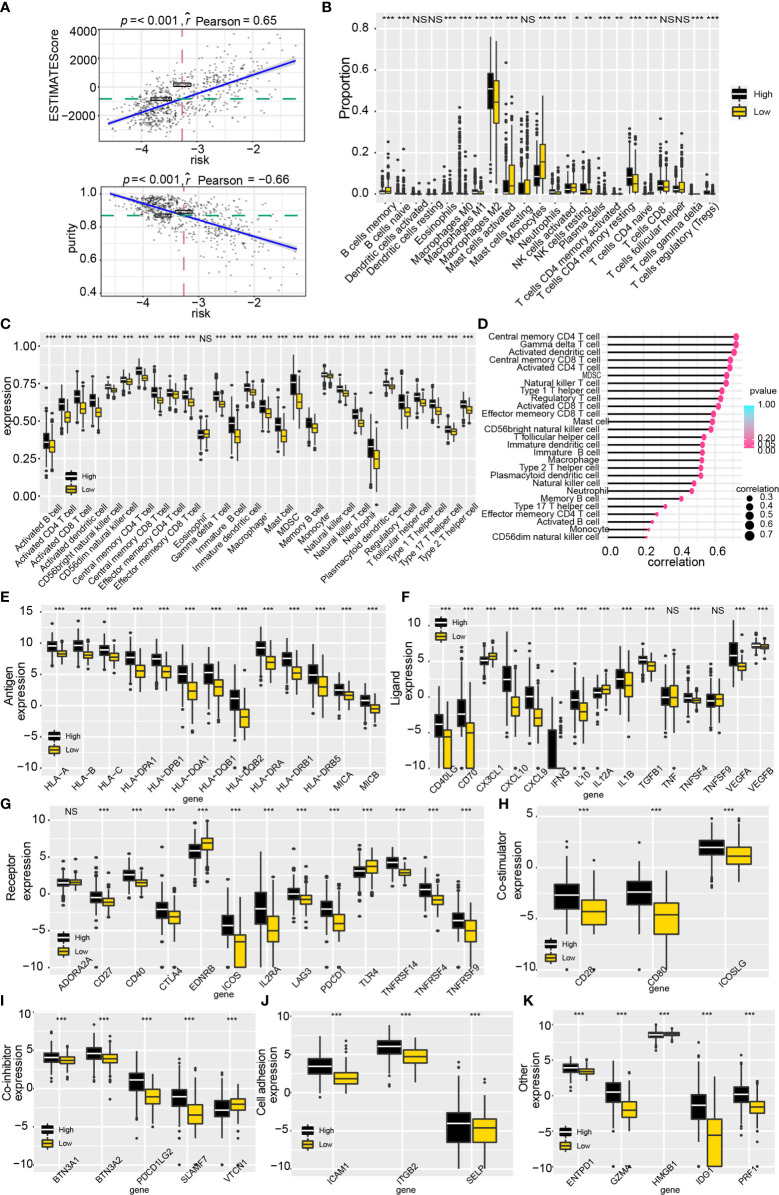
Tumor immune landscape and the expression of immune checkpoint genes based on the scoring system. **(A)** Tumor purity and estimate score were calculated by the ESTIMATE algorithm. **(B)** The proportion of immunocytes based on the CIBERSORT algorithm. **(C)** The enrichment score profile of 28 immunocytes. **(D)** Correlation between 28 immunocytes enrichment score and risk. **(E–K)** The expression profile of immune checkpoint genes in the risk model. NS, no significantly statistical; *P < 0.05; **P < 0.01; ***P < 0.001.

First, tumor purity was calculated by applying the ESTIMATE algorithm to evaluate the immunocytes infiltration ratio in high and low-risk groups. The increased estimate score (a combination of stromal score and immune score) and decreased tumor purity in high-risk score samples from TCGA suggested that high-risk score samples possessed a complicated tumor immune landscape with more immunocytes and stromal cells (**Figure 6A**). Results from the validation datasets also drew similar conclusions (**Supplementary Figure 6A**). The composition of immunocytes in the high and low-risk groups was generated by applying the CIBERSORT algorithm and 28-immune cell lineage analysis in the training (**Figures 6B, C**) and validation datasets (**Supplementary Figures 6B, C**). Besides, the correlation between the enrichment score of immunocytes and risk score was also calculated (**Figure 6D** and **Supplementary Figure 6D**). Results showed that the proportion of immunocytes varied significantly between high and low-risk groups. The high-risk group contained more M2 macrophages, regulatory T cells and CD4 memory resting T cells, whereas activated mast cells were relatively lower.

Interestingly, significantly infiltration of immunotherapy-related immunocytes was found in the high-risk group, including activated CD8 T cells, regulatory T cells, helper T cells et al. Accordingly, we explored the expression of tumor immune checkpoint genes (ICGs) for a better understanding of the role of ALDHs in immunotherapy (**Figures 6E–K**). As illustrated, higher expression of ICGs, such as CD40LG, CD40, CTLA4, PDCD1, PDCD1LG2, ICAM1, CD80 and GZMA, was found in the high-risk group, compared to the low-risk group. In summary, ALDHs could participate in reshaping the tumor immune landscape and weakening immunocyte function.

### ALDH3B1 and ALDH16A1 Promote Proliferation and Migration of Glioma Cells

**Figure 7:**
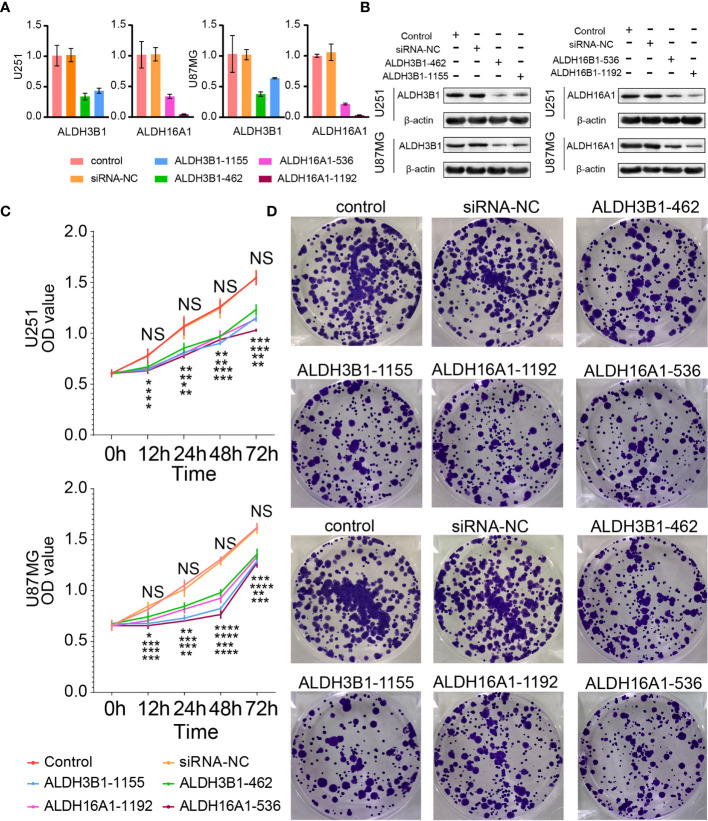
Silenced ALDH3B1 or ALDH16A1 expression inhibits glioma cells progression. The expression of ALDH3B1 was silenced by siRNA-462, siRNA-1155, while ALDH16A1 was silenced by siRNA-536, siRNA-1192. The effect of ALDH depletion by siRNA transfection was detected by qRT-PCR **(A)** and Western blot **(B)**. **(C)** Cell proliferation was measured at the indicated time points by CCK8 assay. **(D)** Representative images of colony formation. All experiments have been independently replicated three times. NS, no significantly statistical; *P < 0.05; **P < 0.01; ***P < 0.001; ****P < 0.0001. n=3. Data are represented as mean ± SD.

Three out of the five components of our risk score, ALDH3A1, ALDH3B1 and ALDH16A1, were identified as tumor promoters. A previous study reported that ALDH3A1 could modulate glioma proliferation and migration (30), whereas the other two genes have been poorly investigated. Therefore, we silenced the expression of ALDH3B1 or ALDH16A1 in two GBM cell lines, U251 and U87MG, to verify their role. RT-qPCR and Western-blotting assay were performed to detect alterations of ALDH3B1 and ALDH16A1 expression. We observed that both mRNA and protein levels of ALDH3B1 and ALDH16A1 were significantly decreased in the silenced group (**Figures 7A, B**). Furthermore, the CCK8 assays showed that the proliferation ratio of cells with silenced ALDH3B1 or ALDH16A1 was significantly reduced (**Figure 7C**). The colony-forming ability of tumor cells was inhibited by decreasing ALDH3B1 and ALDH16A1 expression (**Figure 7D**, statistical results in **Supplementary Figures 8A–D**).

**Figure 8:**
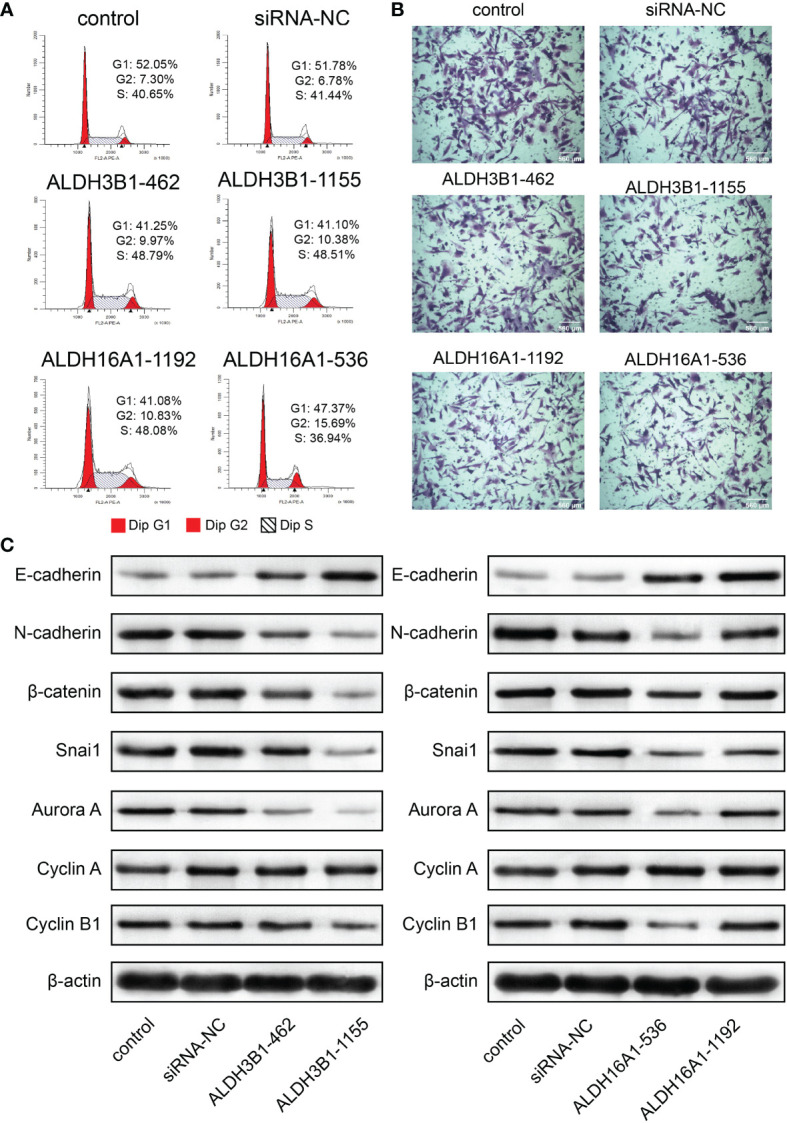
ALDH3B1 and ALDH16A1 affect glioma cells proliferation and migration in U251. The expression of ALDH3B1 was silenced by siRNA-462, siRNA-1155, while ALDH16A1 was silenced by siRNA-536, siRNA-1192. **(A)** Cell cycle was arrested at the G2/M phase by decreasing ALDH3B1 and ALDH16A1 expression. **(B)** Representative images of migration assays for U251 transfected with indicated siRNA. **(C)** The expression of tumor cell cycle and migration associated proteins in U251, including E-cadherin, N-cadherin, β-catenin, snai1, aurora A, cyclin A, cyclin B1 and β-actin. Scale bar = 560μm. All experiments have been independently replicated three times. NS, no significantly statistical; *P < 0.05; **P < 0.01; ***P < 0.001. n=3. Data are represented as mean ± SD.

In addition, flow cytometry results suggested that cells were accumulated at the S phase and G2 phase, implying cell cycle arrest at the G2/M phase (U251 in **Figure 8A**, U87MG in **Figure S7A**). A previous study reported that cyclin A, cyclin B1 and aurora A were G2/M cell cycle checkpoints (31). In this work, cyclin B1 and aurora A expressions were decreased after silenced ALDH3B1 and ALDH16A1 expression; however, no difference in cyclin A expression was observed (U251 in **Figure 8C**, U87MG in **Figure S7C**). Based on these findings, it can be concluded that ALDH3B1 and ALDH16A1 may affect the cell cycle *via* modulation of cyclin B1 and aurora A.

In contrast, the transwell assay showed that tumor cell migration was inhibited by suppressing ALDH3B1 or ALDH16A1 expression (U251 in **Figure 8B**, U87MG in **Supplementary Figure 7B**, statistical results in **Supplementary Figures 8E–H**). E-cadherin expression was elevated while N-cadherin, β-catenin and snai1 expression were decreased, implying that epithelial-mesenchymal transition was inhibited after silencing ALDH3B1 and ALDH16A1 expression (U251 in **Figure 8C**, U87MG in **Supplementary Figure 7C**). Therefore, ALDH3B1 and ALDH16A1 could promote glioma progression by regulating cell proliferation and migration.

## Discussion

Gliomas are the most common primary malignant brain tumors. GBM has been documented with the worst outcomes, with an average survival of less than two years (32). Therefore, it is necessary to explore the underlying biological mechanisms of gliomas to develop new classification systems and design novel treatment approaches (33). The role of ALDHs in gliomas remains elusive; exploring its role may be beneficial in constraining glioma progression.

In this study, we used LASSO regression analysis to construct a risk model of gliomas based on ALDHs. ALDH5A1, ALDH3B1, ALDH3A1, ALDH2 and ALDH16A1 were screened as main contributors of that scoring system to calculate a risk score. Samples with high risk were associated with a worse prognosis. Bioinformatic analysis and *in vitro* experiments suggested that ALDHs influenced the tumor cell cycle, epithelial-mesenchymal transition, and infiltration of immunocytes. Moreover, our constructed prognostic model, nomogram, based on risk and gliomas type, could precisely predict overall survival outcome, indicating its potential clinical application value.

The dysregulated expression of ALDHs has been widely noticed in gliomas and is reported tightly associated with glioma progression. Recently, several studies reported the function of ALDHs in gliomas. An increasing body of evidence suggests that ALDHs target inhibitors could inhibit stem-like cell proliferation of gliomas (18, 34, 35). Furthermore, ALDH1A3 could reportedly affect glioma glucose metabolism and is recognized as a stem-like cells biomarker (36, 37). Besides, ALDH1A1 and ALDH1A2 have been documented as glioma migration-associated genes (38–40), while ALDH1A1 also participated in GBM resistance to temozolomide (17, 19). To the best of our knowledge, this is the first study that identified the role of ALDH16A1 and ALDH3B1 in gliomas.

Based on our scoring system, three out of five ALDHs (ALDH3A1, ALDH3B1 and ALDH16A1) were identified as unfavorable biomarkers for gliomas. Previous studies reported that ALDH3A1 was involved in the Wnt/β-catenin signaling pathway mediated glioblastoma resistance to temozolomide (30). Accordingly, we focused on the role of ALDH3B1 and ALDH16A1 in gliomas.

In the present study, ALDH3B1 and ALDH16A1 were positively correlated with genes involved in cell cycle checkpoint-related pathways and extracellular matrix disassembly-related pathways. Previous studies reported the epithelial-mesenchymal transition as one of the glioma’s malignant factors, suggesting its critical role in glioma progression (41, 42). Importantly, silencing ALDH3B1 and ALDH16A1 expression induced cycle arrest at the G2/M phase and inhibited the epithelial-mesenchymal transition in the glioma cells. Critically, ALDH3B1 and ALDH16A1 could also affect the expression of cyclin B1 and aurora A, G2/M cell cycle checkpoints that modulate glioma cells proliferation. Moreover, the expression of epithelial-mesenchymal transition-associated proteins was affected after interfering with ALDH3B1 and ALDH16A1 expression, leading to increased E-cadherin expression and decreased expression of N-cadherin, β-catenin and snai1.

Interestingly, based on single-cell RNA-seq analysis, we found that the system’s main contributors were mostly expressed in tumor cells, OPC and macrophages. As previously mentioned, the influence of ALDHs on tumor cells and their role in glioma stem-like cells have been established by various studies. Apart from that, distribution map from single cell RNA-seq analysis also suggested that ALDHs may affect the tumor immune landscape by modulating immunocytes. Previous studies reported that elevated ALDHs activity could modulate macrophage polarization (43), and tumor-infiltrating macrophages were positively correlated with glioma cell proliferation and invasion (44). In addition, ALDHs also played a vital role in the induction and function of regulatory T cells (45). Taken together, those findings suggested that ALDHs could affect the tumor immune landscape by regulating the communication between tumor cells and immunocytes and tumor response to the immune system.

Several ALDHs have also been associated with glioma sensitivity to temozolomide (30, 46). In our study, higher expression of genes associated with antigen representation, immunocyte inhibitor or stimulator, ligand-receptor were found in the high-risk group compared to the low-risk group. This finding suggested that immunocytes may also be affected the ALDHs. For instance, GZMA has been reported to trigger adaptive immunity by stimulating dendritic cells (47) and modulates T cell expansion in immunotherapy (48). In the meantime, the elevated expression of ICGs such as PDCD1, CD274, CTLA4 may also modulate tumor response to immunotherapy (49). In a nutshell, ALDHs are vastly involved in determining tumor response to treatment, and targeting them could have huge prospects in improving current treatment efficiency.

In conclusion, this study systematically illustrated the role of ALDHs in the tumorigenesis of gliomas. The expression pattern, prognostic value and potential mechanism of ALDHs were analyzed. A clinical model was constructed to predict clinical outcomes. Moreover, our *in vitro* experiments substantiated that ALDH3B1 and ALDH16A1 could affect tumor cells proliferation and epithelial-mesenchymal transition.

## Data Availability Statement

The original contributions presented in the study are included in the article/**Supplementary Material**. Further inquiries can be directed to the corresponding authors.

## Author Contributions

Writing – original draft: ZYW, YM, and YT. Writing-review & editing: ZYW, YM, ZHW, and ZD. Data curation: ZYW. Formal analysis: ZYW. Validation: YM, YT, and HZ. Visualization: XZ and SF. Methodology: XL, and QC. Project administration: TS and QC. Funding acquisition: QC. Supervision: TS and QC. All authors contributed to the article and approved the submitted version.

## Funding

This work was supported by the National Nature Science Foundation of China (NO.82073893, 81703622); the China Postdoctoral Science Foundation (NO.2018M633002); Hunan Provincial Natural Science Foundation of China (NO.2018JJ3838, 2020JJ8111); and grants from the research projects of Hunan Provincial Health Commission of China (NO.C2019186); Xiangya Hospital Central South University postdoctoral foundation; and the Fundamental Research Funds for the Central Universities of Central South University (No. 2021zzts1027).

## Conflict of Interest

The authors declare that the research was conducted in the absence of any commercial or financial relationships that could be construed as a potential conflict of interest.

## Publisher’s Note

All claims expressed in this article are solely those of the authors and do not necessarily represent those of their affiliated organizations, or those of the publisher, the editors and the reviewers. Any product that may be evaluated in this article, or claim that may be made by its manufacturer, is not guaranteed or endorsed by the publisher.

## References

[1] Ostrom QT Gittleman H Farah P Ondracek A Chen Y Wolinsky Y. CBTRUS Statistical Report: Primary Brain and Central Nervous System Tumors Diagnosed in the United States in 2006-2010. Neuro Oncol (2013) 15(Suppl 2):ii1–56. doi: 10.1093/neuonc/not151 24137015 PMC3798196

[2] Louis DN Ohgaki H Wiestler OD Cavenee WK Burger PC Jouvet A. The 2007 WHO Classification of Tumours of the Central Nervous System. Acta Neuropathol (2007) 114:97–109. doi: 10.1007/s00401-007-0243-4 17618441 PMC1929165

[3] Wick W Osswald M Wick A Winkler F. Treatment of Glioblastoma in Adults. Ther Adv Neurol Disord (2018) 11:1756286418790452. doi: 10.1177/1756286418790452 30083233 PMC6071154

[4] Zhang L Liu F Weygant N Zhang J Hu P Qin Z. A Novel Integrated System Using Patient-Derived Glioma Cerebral Organoids and Xenografts for Disease Modeling and Drug Screening. Cancer Lett (2021) 500:87–97. doi: 10.1016/j.canlet.2020.12.013 33309780

[5] van den Bent MJ. Interobserver Variation of the Histopathological Diagnosis in Clinical Trials on Glioma: A Clinician's Perspective. Acta Neuropathol (2010) 120:297–304. doi: 10.1007/s00401-010-0725-7 20644945 PMC2910894

[6] Molinaro AM Taylor JW Wiencke JK Wrensch MR. Genetic and Molecular Epidemiology of Adult Diffuse Glioma. Nat Rev Neurol (2019) 15:405–17. doi: 10.1038/s41582-019-0220-2 PMC7286557 31227792

[7] Louis DN Perry A Reifenberger G von Deimling A Figarella-Branger D Cavenee WK. The 2016 World Health Organization Classification of Tumors of the Central Nervous System: A Summary. Acta Neuropathol (2016) 131:803–20. doi: 10.1007/s00401-016-1545-1 27157931

[8] Wang Q Hu B Hu X Kim H Squatrito M Scarpace L. Tumor Evolution of Glioma-Intrinsic Gene Expression Subtypes Associates With Immunological Changes in the Microenvironment. Cancer Cell (2017) 32:42–56 e6. doi: 10.1016/j.ccell.2017.06.003 28697342 PMC5599156

[9] Rodriguez-Zavala JS Calleja LF Moreno-Sanchez R Yoval-Sanchez B. Role of Aldehyde Dehydrogenases in Physiopathological Processes. Chem Res Toxicol (2019) 32:405–20. doi: 10.1021/acs.chemrestox.8b00256 30628442

[10] Toledo-Guzman ME Hernandez MI Gomez-Gallegos AA Ortiz-Sanchez E. ALDH as a Stem Cell Marker in Solid Tumors. Curr Stem Cell Res Ther (2019) 14:375–88. doi: 10.2174/1574888X13666180810120012 30095061

[11] Vlashi E Pajonk F. Cancer Stem Cells, Cancer Cell Plasticity and Radiation Therapy. Semin Cancer Biol (2015) 31:28–35. doi: 10.1016/j.semcancer.2014.07.001 25025713 PMC4291301

[12] Zhang W Liu Y Hu H Huang H Bao Z Yang P. ALDH1A3: A Marker of Mesenchymal Phenotype in Gliomas Associated With Cell Invasion. PloS One (2015) 10:e0142856. doi: 10.1371/journal.pone.0142856 26575197 PMC4648511

[13] Wu W Schecker J Wurstle S Schneider F Schonfelder M Schlegel J. Aldehyde Dehydrogenase 1A3 (ALDH1A3) Is Regulated by Autophagy in Human Glioblastoma Cells. Cancer Lett (2018) 417:112–23. doi: 10.1016/j.canlet.2017.12.036 29306018

[14] Li G Li Y Liu X Wang Z Zhang C Wu F. ALDH1A3 Induces Mesenchymal Differentiation and Serves as a Predictor for Survival in Glioblastoma. Cell Death Dis (2018) 9:1190. doi: 10.1038/s41419-018-1232-3 30538217 PMC6290011

[15] Nakano I. Stem Cell Signature in Glioblastoma: Therapeutic Development for a Moving Target. J Neurosurg (2015) 122:324–30. doi: 10.3171/2014.9.JNS132253 25397368

[16] Cheng P Wang J Waghmare I Sartini S Coviello V Zhang Z. FOXD1-ALDH1A3 Signaling Is a Determinant for the Self-Renewal and Tumorigenicity of Mesenchymal Glioma Stem Cells. Cancer Res (2016) 76:7219–30. doi: 10.1158/0008-5472.CAN-15-2860 PMC5161538 27569208

[17] Xu SL Liu S Cui W Shi Y Liu Q Duan JJ. Aldehyde Dehydrogenase 1A1 Circumscribes High Invasive Glioma Cells and Predicts Poor Prognosis. Am J Cancer Res (2015) 5:1471–83. PMC4473324 26101711

[18] Park J Shim JK Kang JH Choi J Chang JH Kim SY. Regulation of Bioenergetics Through Dual Inhibition of Aldehyde Dehydrogenase and Mitochondrial Complex I Suppresses Glioblastoma Tumorspheres. Neuro Oncol (2018) 20:954–65. doi: 10.1093/neuonc/nox243 PMC6007558 29294080

[19] Schafer A Teufel J Ringel F Bettstetter M Hoepner I Rasper M. Aldehyde Dehydrogenase 1A1–A New Mediator of Resistance to Temozolomide in Glioblastoma. Neuro Oncol (2012) 14:1452–64. doi: 10.1093/neuonc/nos270 PMC3499020 23132408

[20] Wang R Sharma R Shen X Laughney AM Funato K Clark PJ. Adult Human Glioblastomas Harbor Radial Glia-Like Cells. Stem Cell Rep (2020) 15:275–7. doi: 10.1016/j.stemcr.2020.06.002 PMC7363934 32668221

[21] Mermel CH Schumacher SE Hill B Meyerson ML Beroukhim R Getz G. GISTIC2.0 Facilitates Sensitive and Confident Localization of the Targets of Focal Somatic Copy-Number Alteration in Human Cancers. Genome Biol (2011) 12:R41. doi: 10.1186/gb-2011-12-4-r41 21527027 PMC3218867

[22] Wang Z Su G Dai Z Meng M Zhang H Fan F. Circadian Clock Genes Promote Glioma Progression by Affecting Tumour Immune Infiltration and Tumour Cell Proliferation. Cell Prolif (2021) 54:e12988. doi: 10.1111/cpr.12988 33442944 PMC7941241

[23] Aibar S Gonzalez-Blas CB Moerman T Huynh-Thu VA Imrichova H Hulselmans G. SCENIC: Single-Cell Regulatory Network Inference and Clustering. Nat Methods (2017) 14:1083–6. doi: 10.1038/nmeth.4463 PMC5937676 28991892

[24] Corridoni D Antanaviciute A Gupta T Fawkner-Corbett D Aulicino A Jagielowicz M. Single-Cell Atlas of Colonic CD8(+) T Cells in Ulcerative Colitis. Nat Med (2020) 26(9):1480–90. doi: 10.1038/s41591-020-1003-4 32747828

[25] Yoshihara K Shahmoradgoli M Martinez E Vegesna R Kim H Torres-Garcia W. Inferring Tumour Purity and Stromal and Immune Cell Admixture From Expression Data. Nat Commun (2013) 4:2612. doi: 10.1038/ncomms3612 24113773 PMC3826632

[26] Newman AM Liu CL Green MR Gentles AJ Feng W Xu Y. Robust Enumeration of Cell Subsets From Tissue Expression Profiles. Nat Methods (2015) 12:453–7. doi: 10.1038/nmeth.3337 PMC4739640 25822800

[27] Charoentong P Finotello F Angelova M Mayer C Efremova M Rieder D. Pan-Cancer Immunogenomic Analyses Reveal Genotype-Immunophenotype Relationships and Predictors of Response to Checkpoint Blockade. Cell Rep (2017) 18:248–62. doi: 10.1016/j.celrep.2016.12.019 28052254

[28] Schreiber RD Old LJ Smyth MJ. Cancer Immunoediting: Integrating Immunity's Roles in Cancer Suppression and Promotion. Science (2011) 331:1565–70. doi: 10.1126/science.1203486 21436444

[29] Wang S Zhang Q Yu C Cao Y Zuo Y Yang L. Immune Cell Infiltration-Based Signature for Prognosis and Immunogenomic Analysis in Breast Cancer. Brief Bioinform (2020) 22(2):2020–31. doi: 10.1093/bib/bbaa026 32141494

[30] Suwala AK Koch K Rios DH Aretz P Uhlmann C Ogorek I. Inhibition of Wnt/beta-Catenin Signaling Downregulates Expression of Aldehyde Dehydrogenase Isoform 3A1 (ALDH3A1) to Reduce Resistance Against Temozolomide in Glioblastoma *In Vitro* . Oncotarget (2018) 9:22703–16. doi: 10.18632/oncotarget.25210 PMC5978259 29854309

[31] Ding L Yang L He Y Zhu B Ren F Fan X. CREPT/RPRD1B Associates With Aurora B to Regulate Cyclin B1 Expression for Accelerating the G2/M Transition in Gastric Cancer. Cell Death Dis (2018) 9:1172. doi: 10.1038/s41419-018-1211-8 30518842 PMC6281615

[32] Stupp R Mason WP van den Bent MJ Weller M Fisher B Taphoorn MJ. Radiotherapy Plus Concomitant and Adjuvant Temozolomide for Glioblastoma. N Engl J Med (2005) 352:987–96. doi: 10.1056/NEJMoa043330 15758009

[33] Dinavahi SS Bazewicz CG Gowda R Robertson GP. Aldehyde Dehydrogenase Inhibitors for Cancer Therapeutics. Trends Pharmacol Sci (2019) 40:774–89. doi: 10.1016/j.tips.2019.08.002 31515079

[34] Park HH Park J Cho HJ Shim JK Moon JH Kim EH. Combinatorial Therapeutic Effect of Inhibitors of Aldehyde Dehydrogenase and Mitochondrial Complex I, and the Chemotherapeutic Drug, Temozolomide Against Glioblastoma Tumorspheres. Molecules (2021) 26(2):282. doi: 10.3390/molecules26020282 PMC7827959 33429981

[35] Quattrini L Gelardi ELM Coviello V Sartini S Ferraris DM Mori M. Imidazo[1,2-A]Pyridine Derivatives as Aldehyde Dehydrogenase Inhibitors: Novel Chemotypes to Target Glioblastoma Stem Cells. J Med Chem (2020) 63:4603–16. doi: 10.1021/acs.jmedchem.9b01910 32223240

[36] Ni W Xia Y Luo L Wen F Hu D Bi Y. High Expression of ALDH1A3 Might Independently Influence Poor Progression-Free and Overall Survival in Patients With Glioma *via* Maintaining Glucose Uptake and Lactate Production. Cell Biol Int (2020) 44:569–82. doi: 10.1002/cbin.11257 31642564

[37] Gan C Pierscianek D El Hindy N Ahmadipour Y Keyvani K Sure U. The Predominant Expression of Cancer Stem Cell Marker ALDH1A3 in Tumor Infiltrative Area Is Associated With Shorter Overall Survival of Human Glioblastoma. BMC Cancer (2020) 20:672. doi: 10.1186/s12885-020-07153-0 32680476 PMC7368792

[38] Flahaut M Jauquier N Chevalier N Nardou K Balmas Bourloud K Joseph JM. Aldehyde Dehydrogenase Activity Plays a Key Role in the Aggressive Phenotype of Neuroblastoma. BMC Cancer (2016) 16:781. doi: 10.1186/s12885-016-2820-1 27724856 PMC5057398

[39] Adam SA Schnell O Poschl J Eigenbrod S Kretzschmar HA Tonn JC. ALDH1A1 Is a Marker of Astrocytic Differentiation During Brain Development and Correlates With Better Survival in Glioblastoma Patients. Brain Pathol (2012) 22:788–97. doi: 10.1111/j.1750-3639.2012.00592.x PMC8057636 22417385

[40] Wang Z Zhang H Xu S Liu Z Cheng Q. The Adaptive Transition of Glioblastoma Stem Cells and Its Implications on Treatments. Signal Transduct Target Ther (2021) 6:124. doi: 10.1038/s41392-021-00491-w 33753720 PMC7985200

[41] Pan CM Chan KH Chen CH Jan CI Liu MC Lin CM. MicroRNA-7 Targets T-Box 2 to Inhibit Epithelial-Mesenchymal Transition and Invasiveness in Glioblastoma Multiforme. Cancer Lett (2020) 493:133–42. doi: 10.1016/j.canlet.2020.08.024 32861705

[42] Tao C Huang K Shi J Hu Q Li K Zhu X. Genomics and Prognosis Analysis of Epithelial-Mesenchymal Transition in Glioma. Front Oncol (2020) 10:183. doi: 10.3389/fonc.2020.00183 32154177 PMC7047417

[43] Raghavan S Mehta P Xie Y Lei YL Mehta G. Ovarian Cancer Stem Cells and Macrophages Reciprocally Interact Through the WNT Pathway to Promote Pro-Tumoral and Malignant Phenotypes in 3D Engineered Microenvironments. J Immunother Cancer (2019) 7:190. doi: 10.1186/s40425-019-0666-1 31324218 PMC6642605

[44] Quail DF Bowman RL Akkari L Quick ML Schuhmacher AJ Huse JT. The Tumor Microenvironment Underlies Acquired Resistance to CSF-1R Inhibition in Gliomas. Science (2016) 352:aad3018. doi: 10.1126/science.aad3018 27199435 PMC5450629

[45] Bazewicz CG Dinavahi SS Schell TD Robertson GP. Aldehyde Dehydrogenase in Regulatory T-Cell Development, Immunity and Cancer. Immunology (2019) 156:47–55. doi: 10.1111/imm.13016 30387499 PMC6283653

[46] Wen YT Wu AT Bamodu OA Wei L Lin CM Yen Y. A Novel Multi-Target Small Molecule, LCC-09, Inhibits Stemness and Therapy-Resistant Phenotypes of Glioblastoma Cells by Increasing miR-34a and Deregulating the DRD4/Akt/mTOR Signaling Axis. Cancers (Basel) (2019) 11(10):1442. doi: 10.3390/cancers11101442 PMC6826618 31561595

[47] Shimizu K Yamasaki S Sakurai M Yumoto N Ikeda M Mishima-Tsumagari C. Granzyme A Stimulates pDCs to Promote Adaptive Immunity *via* Induction of Type I IFN. Front Immunol (2019) 10:1450. doi: 10.3389/fimmu.2019.01450 31293597 PMC6606709

[48] Inoue H Park JH Kiyotani K Zewde M Miyashita A Jinnin M. Intratumoral Expression Levels of PD-L1, GZMA, and HLA-A Along With Oligoclonal T Cell Expansion Associate With Response to Nivolumab in Metastatic Melanoma. Oncoimmunology (2016) 5:e1204507. doi: 10.1080/2162402X.2016.1204507 27757299 PMC5048759

[49] Zhang H Dai Z Wu W Wang Z Zhang N Zhang L. Regulatory Mechanisms of Immune Checkpoints PD-L1 and CTLA-4 in Cancer. J Exp Clin Cancer Res (2021) 40:184. doi: 10.1186/s13046-021-01987-7 34088360 PMC8178863

