# Supplementary material for: The ALDH Family Contributes to Immunocyte Infiltration, Proliferation and Epithelial-Mesenchymal Transformation in Glioma
Source: Front Immunol. 2022 Jan 7;12:756606. doi: 10.3389/fimmu.2021.756606 (PMC8805082; doi:10.3389/fimmu.2021.756606)
Supplement: Supplementary file 1 [file Presentation_1.pdf]

Supplementary table 1 | Details about the training cohort and validation cohort.

Databases	Cancer (LGG: GBM)	WHO grade (II: III: IV)	IDH (Wildtype: Mutant)	1p19q (Codel: Non-codel)	MGMT (Methylated: Unmethylated)	Gender (Male: Female)
TCGA	522:150	256: 265: 150	228: 434	171: 497	478: 157	387: 185
CGGA1	180:136	102:78:136	148: 172	67: 250	154: 147	199:121
CGGA2	173:124	116: 57: 124	165: 133	16: 76	98: 187	179: 121
GSE108474	170: 124	86: 82: 124	/	/	/	/

Supplementary table 2 | Univariate cox analysis of ALDHs.

Gene	HR	95%CI	P-value
ALDH16A1	2.068	1.764-2.424	2.91E-19
ALDH18A1	0.793	0.637-0.987	0.037764
ALDH1A1	0.866	0.795-0.942	0.000867
ALDH1A2	1.131	1.018-1.256	0.022339
ALDH1A3	1.205	1.149-1.263	1.91E-14
ALDH1B1	0.78	0.616-0.986	0.037825
ALDH1L1	1.136	1.014-1.272	0.027157
ALDH1L2	0.625	0.564-0.692	1.40E-19
ALDH2	0.473	0.421-0.531	7.58E-37
ALDH3A1	1.399	1.315-1.488	1.93E-26
ALDH3A2	1.264	0.997-1.601	0.052665
ALDH3B1	1.663	1.458-1.897	3.18E-14
ALDH4A1	0.917	0.807-1.041	0.180713
ALDH5A1	0.483	0.439-0.532	2.08E-49
ALDH6A1	0.574	0.511-0.645	1.21E-20
ALDH7A1	1.229	1.081-1.397	0.001645
ALDH8A1	0.88	0.802-0.966	0.007245
ALDH9A1	0.788	0.615-1.009	0.059289

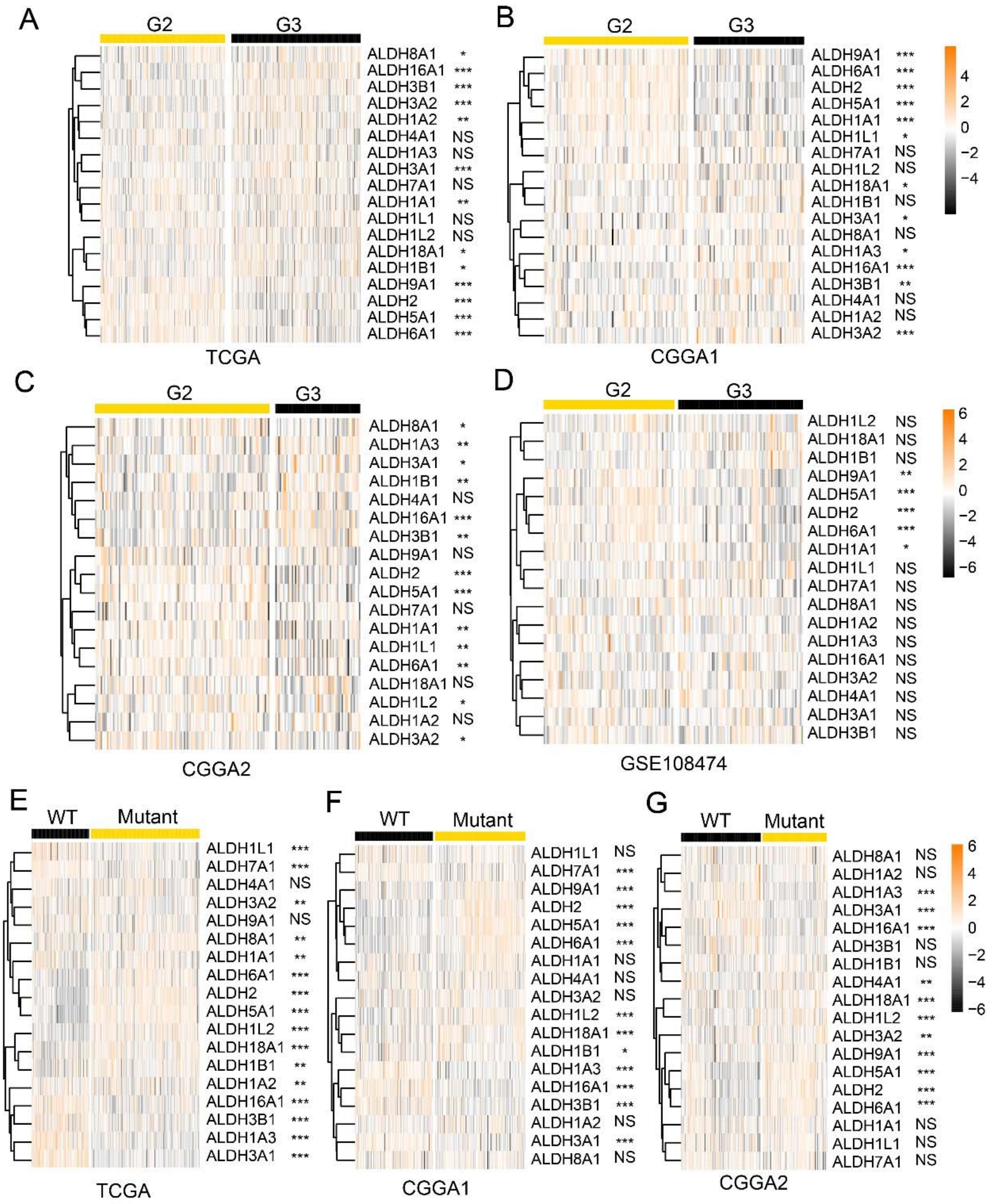

Supplementary Figure 1 | The expression profile of ALDHs. ALDHs expression difference between WHO grade II gliomas and WHO grade III gliomas in the TCGA (A), CGGA1 (B), CGGA2 (C) and GSE108474 (D) databases. The expression profile of ALDHs with different IDH status in the TCGA (E), CGGA1 (F) and CGGA2 (G) datasets. NS: no significantly statistical; *P < 0.05; **P < 0.01; ***P < 0.001.

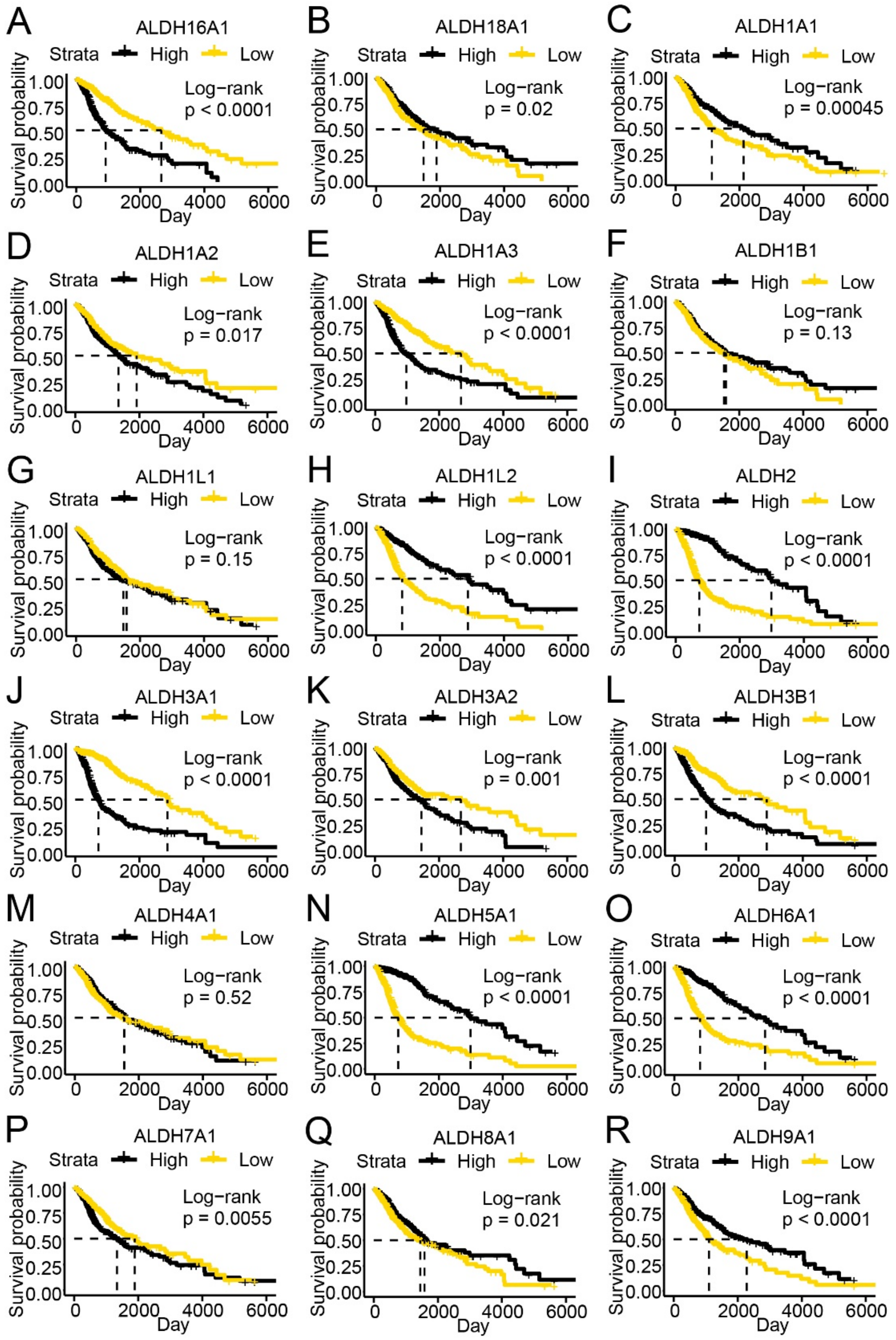

Supplementary Figure 2 | The overall survival analysis of ALDHs. The overall survival analysis based on ALDH16A1 (A, $P < 0.0001$), ALDH18A1 (B, $P = 0.02$), ALDH1A1 (C, $P = 0.00045$), ALDH1A2 (D, $P = 0.017$), ALDH1A3 (E, $P < 0.0001$), ALDH1B1 (F, $P = 0.13$), ALDH1L1 (G, $P = 0.15$), ALDH1L2 (H, $P < 0.0001$), ALDH2 (I, $P < 0.0001$), ALDH3A1 (J, $P < 0.0001$), ALDH3A2 (K, $P = 0.001$), ALDH3B1 (L, $P < 0.0001$), ALDH4A1 (M, $P = 0.52$), ALDH5A1 (N, $P < 0.0001$), ALDH6A1 (O, $P < 0.0001$), ALDH7A1 (P, $P = 0.0055$), ALDH8A1 (Q, $P = 0.021$), ALDH9A1 (R, $P < 0.0001$).

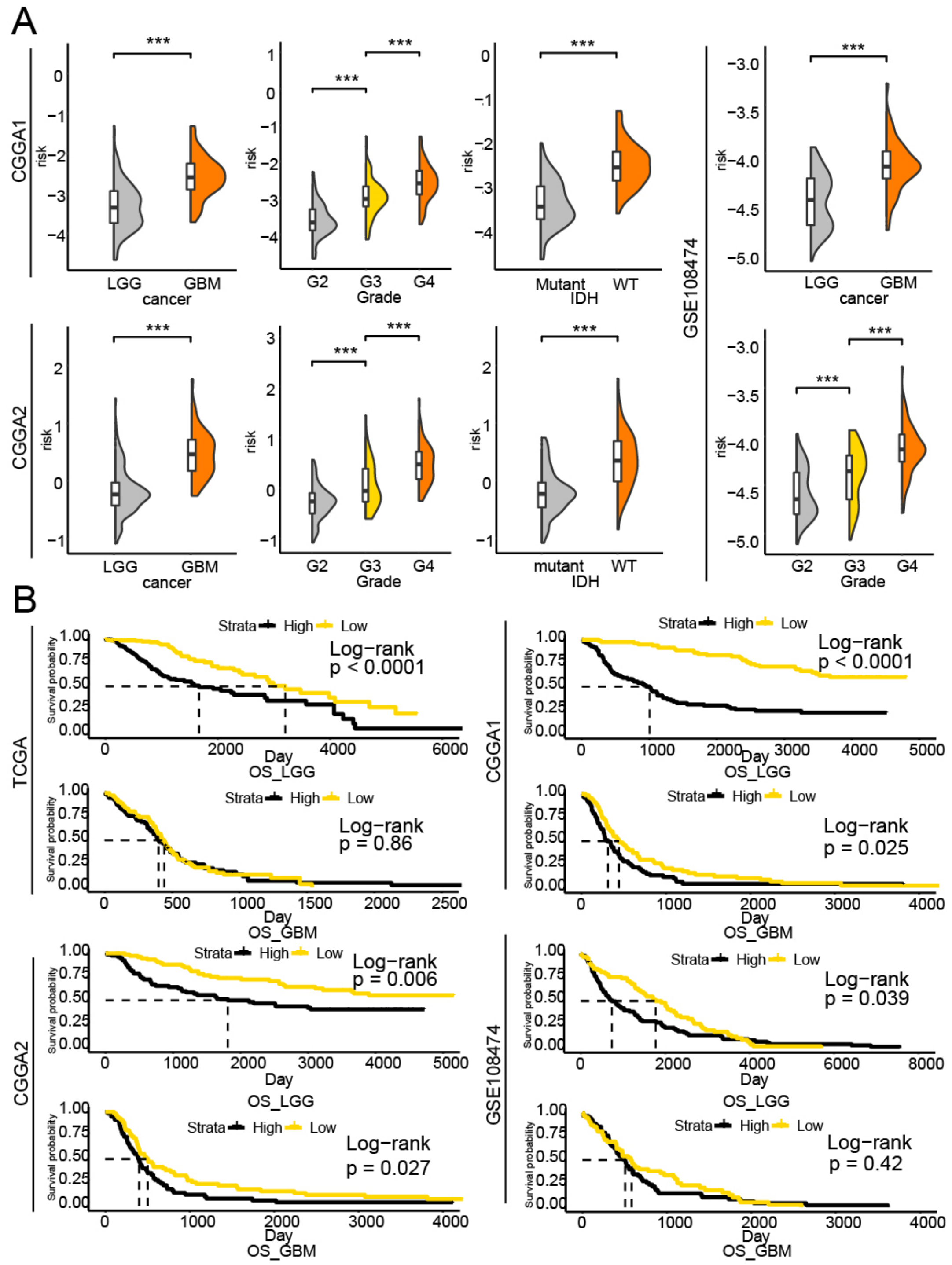

Supplementary Figure 3 | The scoring system based on the ALDH family. (A) The distribution of risk based on cancer type, WHO grades and the IDH status in the CGGA1, CGGA2 and GSE108474 datasets. (B) Overall survival analysis based on risk in the LGG or GBM cohort from the training cohort and validation cohort. NS: no significantly statistical; *P < 0.05; **P < 0.01; ***P < 0.001.

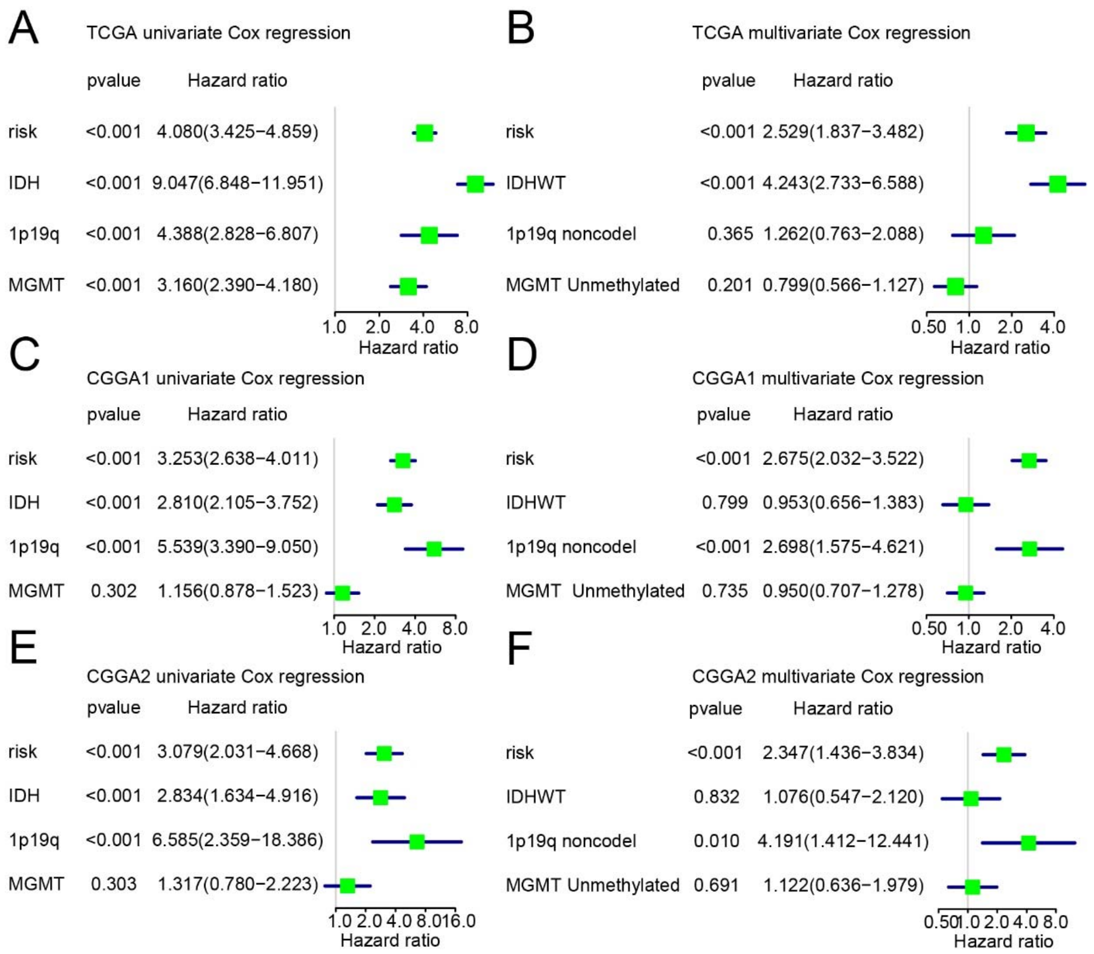

Supplementary Figure 4 | Univariate and multivariate Cox regression analysis. Four factors, risk, the IDH status, the 1p19a status and the MGMT status, were enrolled for univariate and multivariate Cox regression analysis in TCGA (A-B), CGGA1 (C-D) and CGGA2 (E-F).

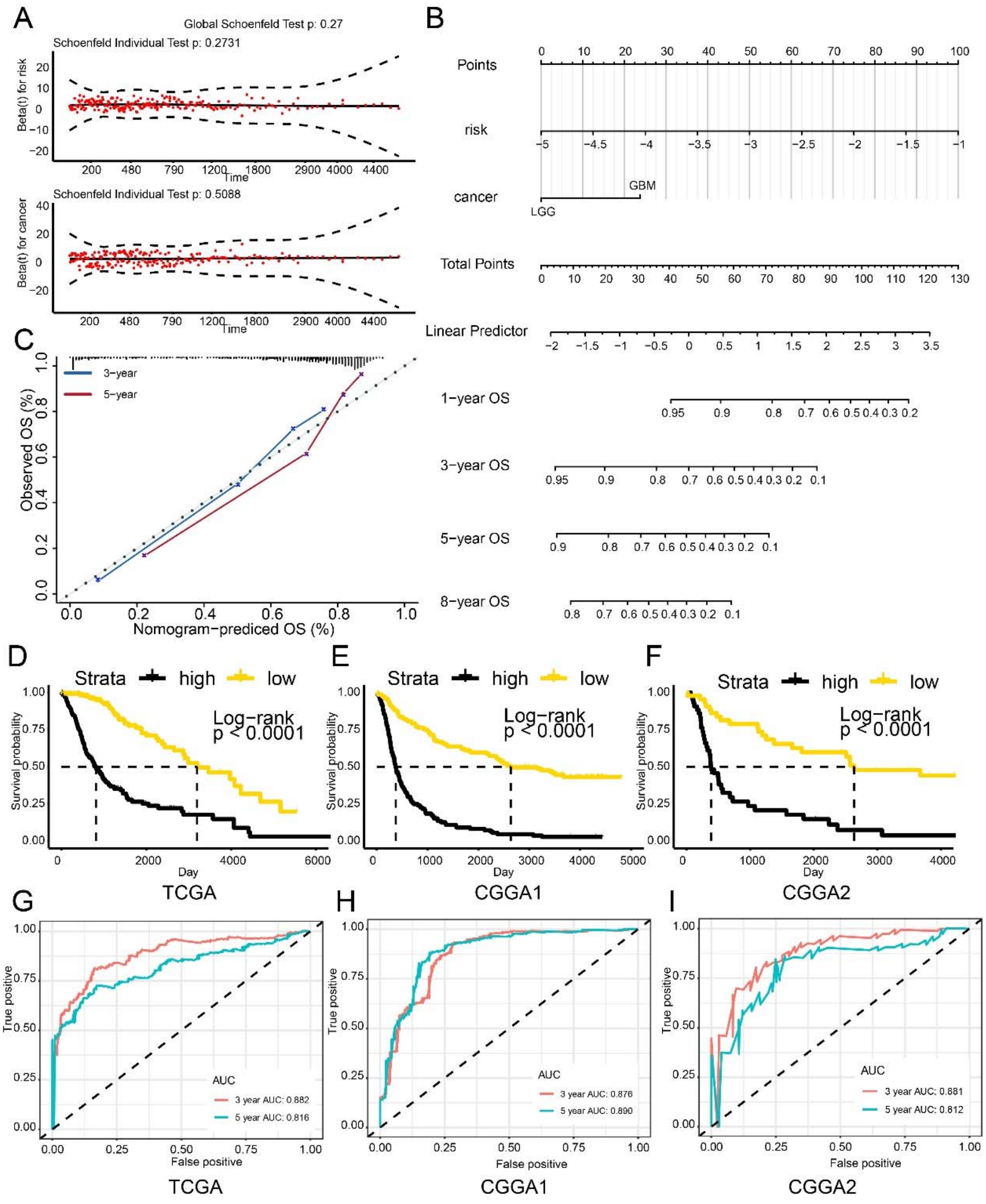

Supplementary Figure 5 | Nomogram based on risk and cancer type. (A) The schoenfeld residual test of factors involved in nomogram. (B) Nomogram to predict OS in glioma patients was created based on risk score and cancer type. (C) The calibration curves of the nomogram to predict OS at 3 years and 5 years. Kaplan-Meier curves for patients according to nomogram scores in the TCGA (D), CGGA1 (E), CGGA2 (F) datasets. ROC curves of the nomogram in the TCGA (G), CGGA1 (H) CGGA2 (I) datasets.

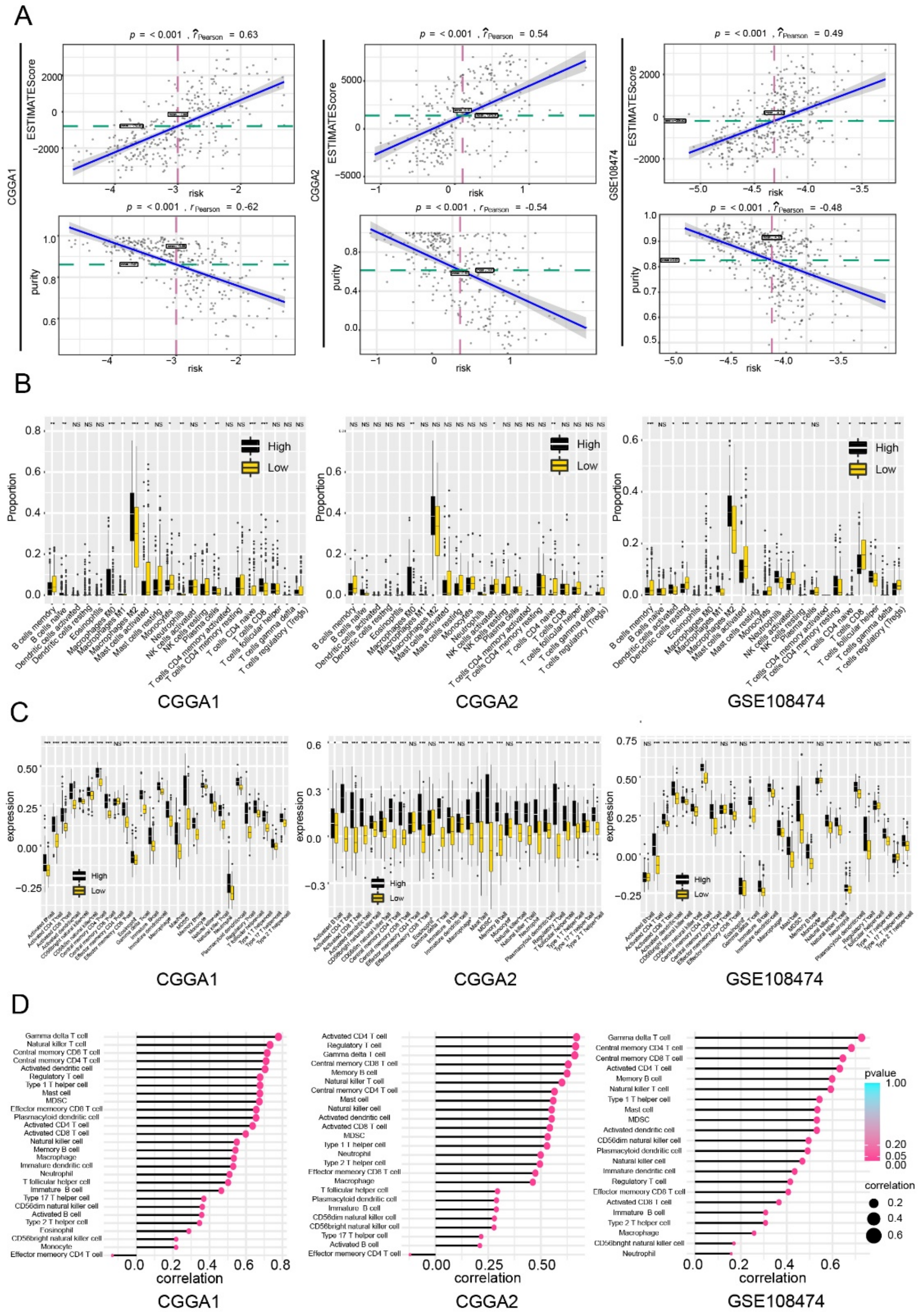

Supplementary Figure 6 | Tumor immune landscape difference according to the risk score model. (A) The ESTIMATE algorithm based on CGGA1, CGGA2 and GSE108474 datasets. (B) Tumor immunocytes infiltration based on the CIRBERSCORT algorithm in the CGGA1, CGGA2 and GSE108474 datasets. (C) Distribution of the enrichment score of 28 immunocytes based on the risk score model in the CGGA1, CGGA2 and GSE108474 databases. (D) Correlation between enrichment score of 28 subpopulations of immunocytes and risk score in the CGGA1, CGGA2 and GSE108474 databases. NS: no significantly statistical; *P < 0.05; **P < 0.01; ***P < 0.001.

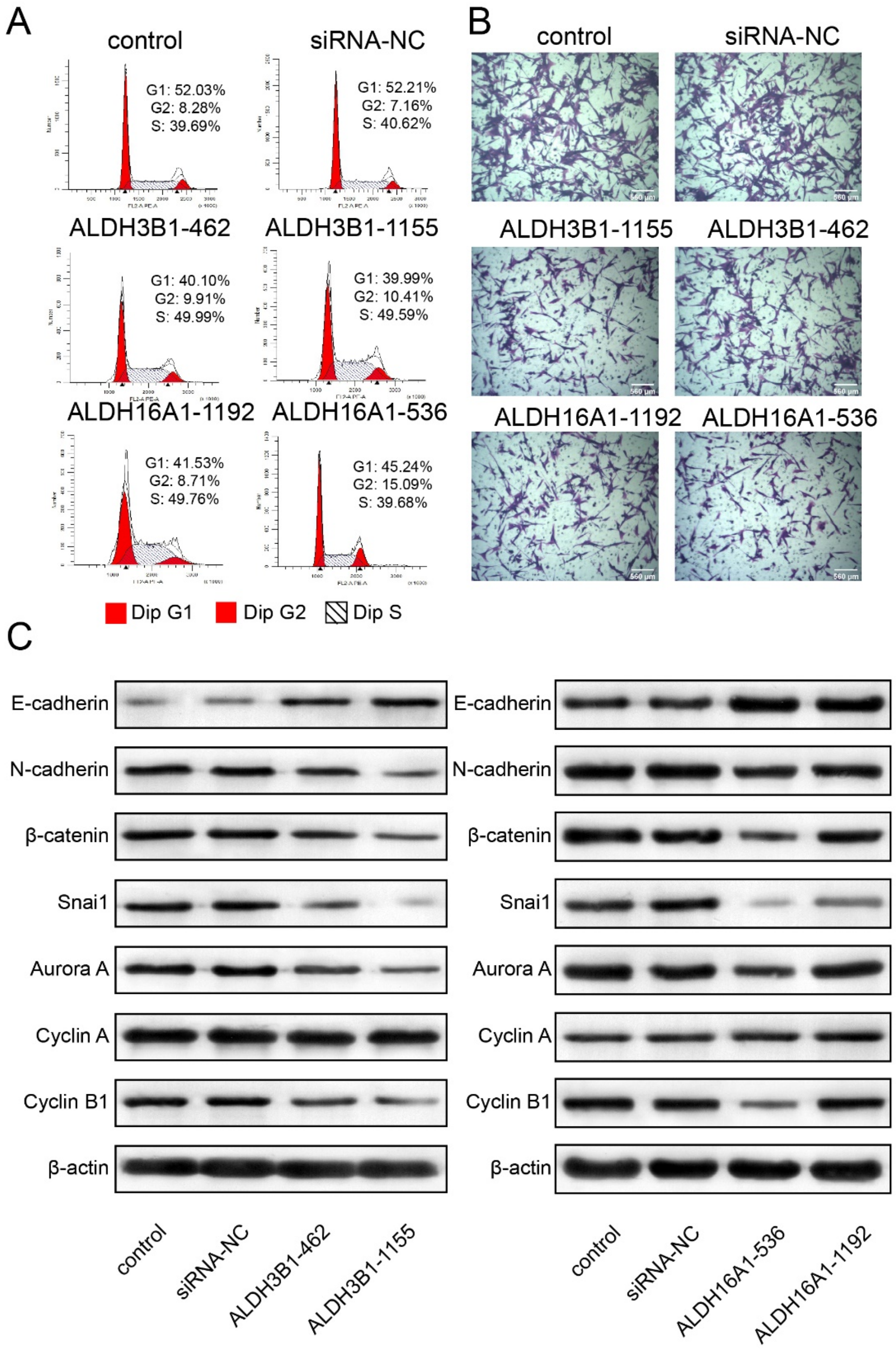

Supplementary Figure 7 | ALDH3B1 and ALDH16A1 affect glioma cells proliferation and migration in U87MG. (A) Cell cycle was arrested at the G2/M phase by decreasing ALDH3B1 and ALDH16A1 expression. (B) Representative images of migration assays for U87MG cells transfected with indicated siRNA, and statistical analysis are shown. (C) The expression of tumor cell cycle and migration associated proteins in U87MG, including E-cadherin, N-cadherin, β-catenin, snai1, aurora A, cyclin A, cyclin B1 and β-actin. Scale bar = 560μm. All experiments have been independently replicated three times. NS: no significantly statistical; *$P < 0.05$; **$P < 0.01$; ***$P < 0.001$. n=3. Data are represented as mean ± SD.

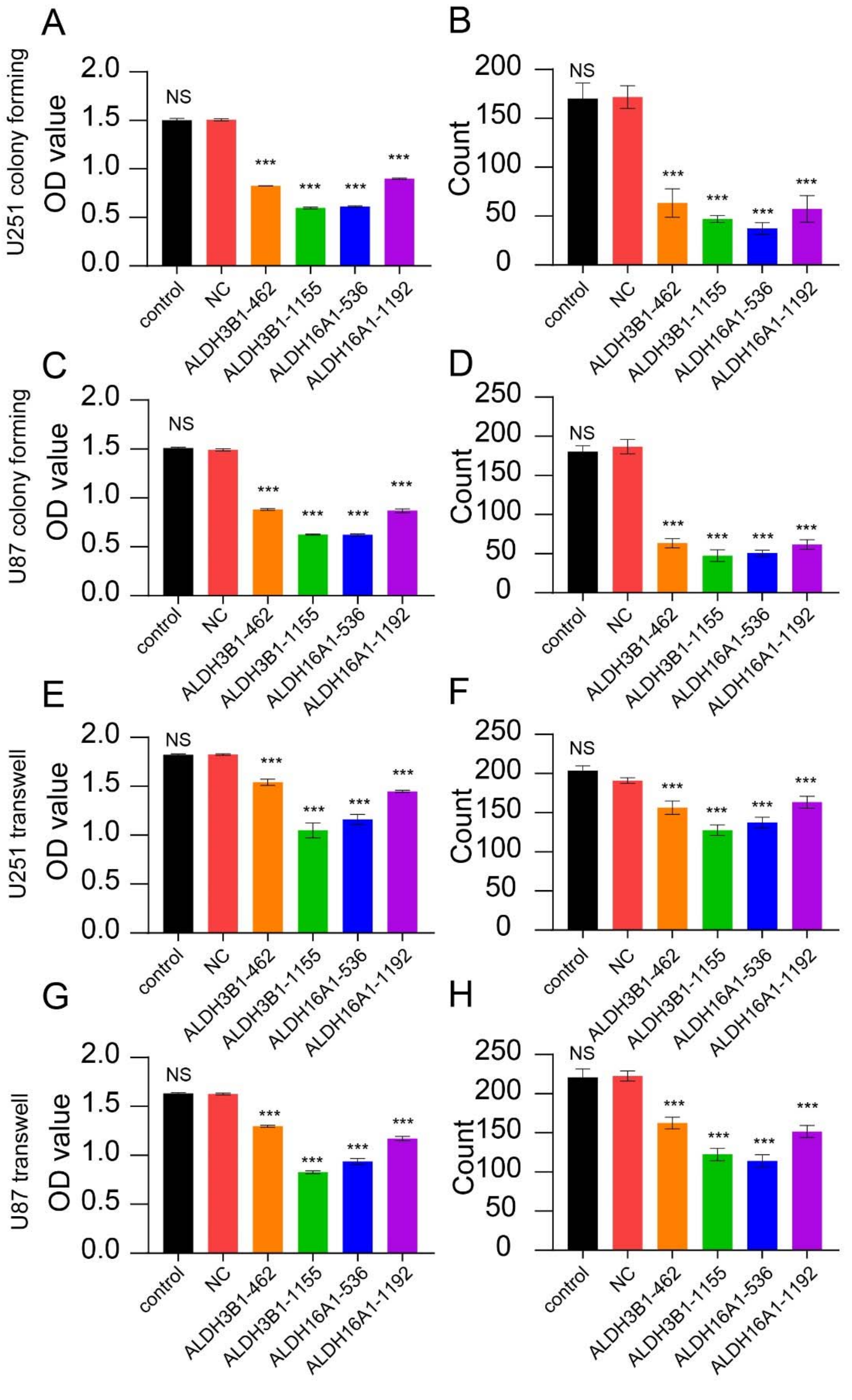

Supplementary Figure 8 | Statistical analysis of the colony forming assay and the transwell assay. OD value (A) and cell counting (B) of the colony forming assay of U251. OD value (C) and cell counting (D) of the colony forming assay of U87MG. OD value (E) and cell counting (F) of the transwell assay of U251. OD value (G) and cell counting (H) of the transwell assay of U87MG. NS: no significantly statistical; *P < 0.05; **P < 0.01; ***P < 0.001. n=3. Data are represented as mean ± SD.
